# High Durability Sliding TENG with Enhanced Output Achieved by Capturing Multiple Region Charges for Harvesting Wind Energy

**DOI:** 10.1007/s40820-025-02043-1

**Published:** 2026-01-07

**Authors:** Wencong He, Yunchuan Liu, Junhao Jin, Jiahao Cai, Buyong Wan, Jie Chen, Xiaohong Yang, Chenguo Hu

**Affiliations:** 1 College of Physics and Electronic Engineering, Chongqing Normal University, Chongqing, 401331 People’s Republic of China; 2 School of Physics, Chongqing Key Laboratory of Soft Condensed Matter Physics and Smart Materials, Chongqing University, Chongqing, 400044 People’s Republic of China

**Keywords:** Triboelectric, Volume effect, Electrostatic breakdown, Durability, Wind energy

## Abstract

Improving the electric output and durability of triboelectric nanogenerator (TENG) remains a great challenge. In sliding-mode TENG, surface charge dissipation and charge leakage caused by the volume effect result in serious energy waste. In this work, a durable dual output mode TENG (DDO-TENG), which includes alteranting current and direct current output modes, is designed to capture the dissipating charges in the surface of charge space accumulation area and the inner leakage charge in porous network to further improve the output performance of sliding TENGs. The output charge density of DDO-TENG reaches 0.847 mC m^−2^, which is 2.39 times as that of the single mode device. In addition, it has strong durability, remaining 95.7% after over 271 k cycles, and it can continuously power electronics by harvesting wind energy. This work provides a strategy for achieving the improvement on output performance and durability and expands the application of TENG.

**Supplementary Information:**

The online version contains supplementary material available at 10.1007/s40820-025-02043-1.

## Highlights

- A dual output mode triboelectric nanogenerator for capturing multiple regions charges is proposed.
- Achieving a 139% improvement in charge transferring rate compared to traditional device.
- A charge density of 846.7 μC m^−2^ is achieved based on microscale dielectric material.
- The device can supply power for remote road signs under wind energy.

**Supplementary Information:**

The online version contains supplementary material available at 10.1007/s40820-025-02043-1.

## Graphical abstract

**Figure:**
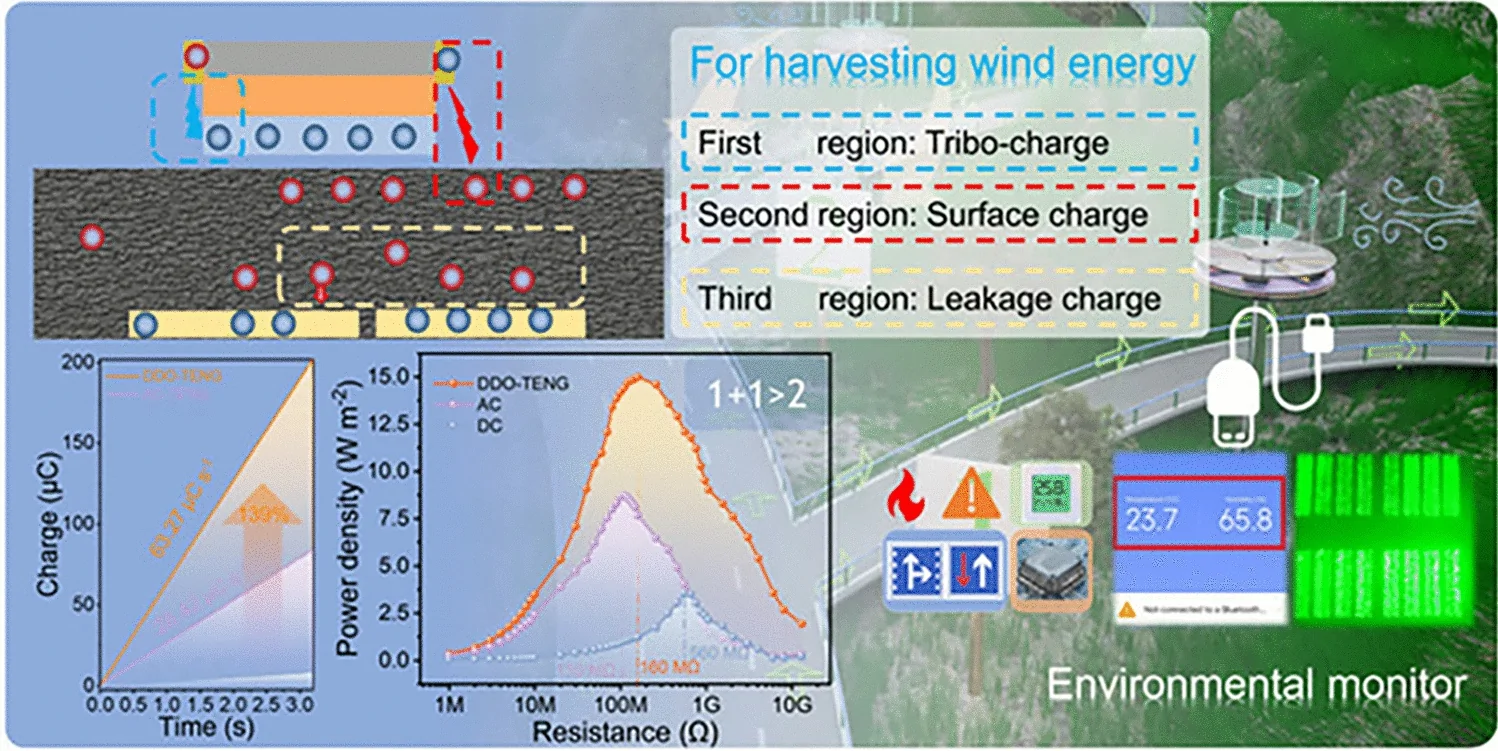

## Introduction

As the social development, the era of artificial intelligence and the Internet of Things has arrived. Traditional sensors are powered by batteries and wire transmission [1]. However, maintaining the batteries of large-scale sensor systems incurs high labor costs, and the disposal of waste batteries is costly. Moreover, the use of wired power sources in outdoor areas is restricted [2–5]. For example, if the electrical energy required by outdoor intelligent transportation systems is transmitted only through cables, the cost will be extremely high, therefore, exploring new power supply model is urgent [6, 7]. Obtaining mechanical energy from the surrounding environment and converting it into electrical energy to ensure the continuous and stable operation of electronic devices is a viable solution [8–10]. Triboelectric nanogenerators as an innovative energy conversion device, can efficiently convert environmental mechanical energy into electrical energy [11–13]. It is low cost [14, 15], lightweight [16, 17], easy to manufacture [18, 19], and has excellent performance at low frequencies [20, 21]. It has very good prospects for development as one of the potential technologies for powering electronic products [22–26].

Efficient energy conversion, high charge density and stable output are the prerequisites for the practical application of triboelectric nanogenerators (TENGs) [27–29]. TENG operates based on the coupling effect of triboelectrification and electrostatic induction. Its output performance is closely related to factors such as the contact area of the triboelectric materials, the friction frequency, and the surface charge density. When some strategies are taken in order to increase the output power, but this will lead to a decrease in the stability of TENG. To address this issue, researchers have proposed strategies such as using a vacuum environment [30, 31], temperature difference [32, 33], surface modification [34–36], charge excitation [37] and charge spatial accumulation [38] to increase their charge density. In recent years, Fu et al. have used materials with strong charge transfer characteristics at the millimeter level as dielectric layer materials, transforming the surface effects of triboelectrification and electrostatic induction into volume effects effectively improves the problem of surface wear of the dielectric material, enhances the stability of TENG, and also achieves a certain improvement in its output [39]. As a result, the charge density of TENG have reached 370 μC m^−2^. However, due to the frictional electricity on the surface, there are still some electric charges that not be converted in time, and then there is an electrostatic air breakdown effect with the air [40], resulting in the dissipation of the charge and reducing part of the power of TENG. Later, Shan et al. designed a direct current triboelectric nanogenerator [41]. By adjusting the moving direction of the slider and the electronegativity difference between two dielectric materials to control the current direction, a unidirectional sliding with bidirectional and dual channel output was achieved, with an average power density of 3 W m^−2^. However, during operation the dielectric materials need to be tightly coupled, and high friction is used in exchange for high output, which seriously affects durability.

In this work, aiming at the problem of insufficient surface charge density and charge transfer efficiency of the dielectric materials in traditional TENGs, the durable dual output mode TENG (DDO-TENG) based on millimeter-thick dielectric film was designed. And a systematic study was carried out on the charge migration efficiency in thick dielectric materials and the influence of electrostatic air breakdown on the charge transport behavior. DDO-TENG can transform the electrostatic induction effect of the surface charges effect of dielectric materials into volume effect and allow charge migration inside the materials, which can not only effectively improve the charge transfer efficiency, but also significantly extend the working duration of the device. Besides, based on the electrostatic breakdown mechanism, DDO-TENG can collect the triboelectric charges that have not been completely transferred due to the volume effect, further improving the charge transfer efficiency. The DDO-TENG achieves a charge density of 847.6 µC m^−2^ and a peak power density of 15 W m^−2^, respectively. Compared with the charge density of previous similar sliding TENGs, a significant improvement has been achieved [39, 42–47]. Under a wind speed of 4 m s^−1^, the wind-driven rotating DDO-TENG can charge a 9.4 mF capacitor to 3.65 V in 80 s with a charging rate of 457 μC s^−1^. Besides, after 271,800 cycles durability test, the output of the DDO-TENG remains stable at above 95.7%, and there is no macroscopical wear on the surface of the material. The DDO-TENG can power various electronic devices, such as miniature sensors and road marking devices. This work provides a new strategy for improving the output performance and durability of TENG in the practical application.

## Experimental Section

### Fabrication of the Sliding-Mode DDO-TENG

The structural design of DDO-TENG employs a modular integration scheme, consisting of a stator module and a sliding module. The stator module utilizes an acrylic substrate with dimensions of 120 × 60 mm^2^ as the base material, two Al films with a 3.5 mm gap, each 35 × 60 mm^2^ and 15 μm in thickness, are symmetrically pasted on its surface as AC output electrodes. A 1 mm thick polyurethane (PU) foam matching the substrate size is then laminated over the electrode layer to serve as the stator tribolayer. The slider is constructed using a layered stacking process, first, an acrylic substrate with dimensions of 35 × 60 mm^2^ is fabricated via laser cutter. An equal sized black foam buffer layer is adhered to the substrate surface, followed by lamination of a 35 × 60 mm^2^ polytetrafluoroethylene (PTFE) film with a thickness of 180 μm as the sliding tribolayers. Two Cu electrodes with 60 × 4 mm^2^ and a thickness of 10 μm are symmetrically positioned on both sides of the slider to enable signal extraction act as CCE. During the parametric study of electrode width variations, the effective width of the sliding module dynamically matches that of the stator electrodes.

### Fabrication of the Rotary DDO-TENG

The rotational TENG employs a double disk structural design, consisting of a fixed stator and a rotating rotor assembly. The stator is based on a 4 mm thick acrylic substrate with an external dimension of 18 × 18 cm^2^ and a central bearing mounting hole. On the substrate surface, six groups of alternating radial Al electrode arrays are deposited, with each electrode pair featuring an outer radius of 8 cm, inner radius of 2 cm, individual electrode central angle of 22°, circumferential gap of 2°, and adjacent electrode group blank area accounting for 14° central angle. Following electrode fabrication, a 90 mm radius 1 mm thick PU foam as the tribolayer. The rotor comprises two components, the main body is a 180 mm diameter acrylic disk with a central bearing hole. The driven component is an inner and outer diameter of 20 and 80 mm, and 22° central angle sector structure composed of acrylic substrate, high density foam buffer layer, and PTFE film. The substrate is laser-cut to shape, with the foam layer precisely matching the substrate dimensions, and the outermost layer being 180 μm thick PTFE film. For electrical connectivity, a copper electrode are symmetrically positioned on both sides of the sector slider to enable signal acquisition during rotational motion.

### Electrical Measurement and Characterization

All devices are mounted on an optical platform (ZPT-G-M-15–10) for measurements. Horizontal sliding motion are driven by a linear motor (FSK40E800-10C7-BC-B57). Rotational motion was executed using a commercial programmable stepper motor (86HSE12N) and controller (TC5510). Short circuit current, transferred charge, and capacitor voltage are measured with an electrometer (Keithley 6514), in conjunction with an NI data acquisition card (USB-6346). While load voltage is determined via a series resistor voltage division method. For wind energy driven device, the fan blades are designed and constructed from acrylic sheets, with blades driven by a centrifugal fan whose rotational speed was controlled by adjusting the input voltage. In mechanism exploration experiments, external excitation voltages are provided by a function generator (UTG2025A) and high voltage amplifier (ATA-7050).

## Results and Discussion

### Structural Design and Working Principle

**Fig. 1:**
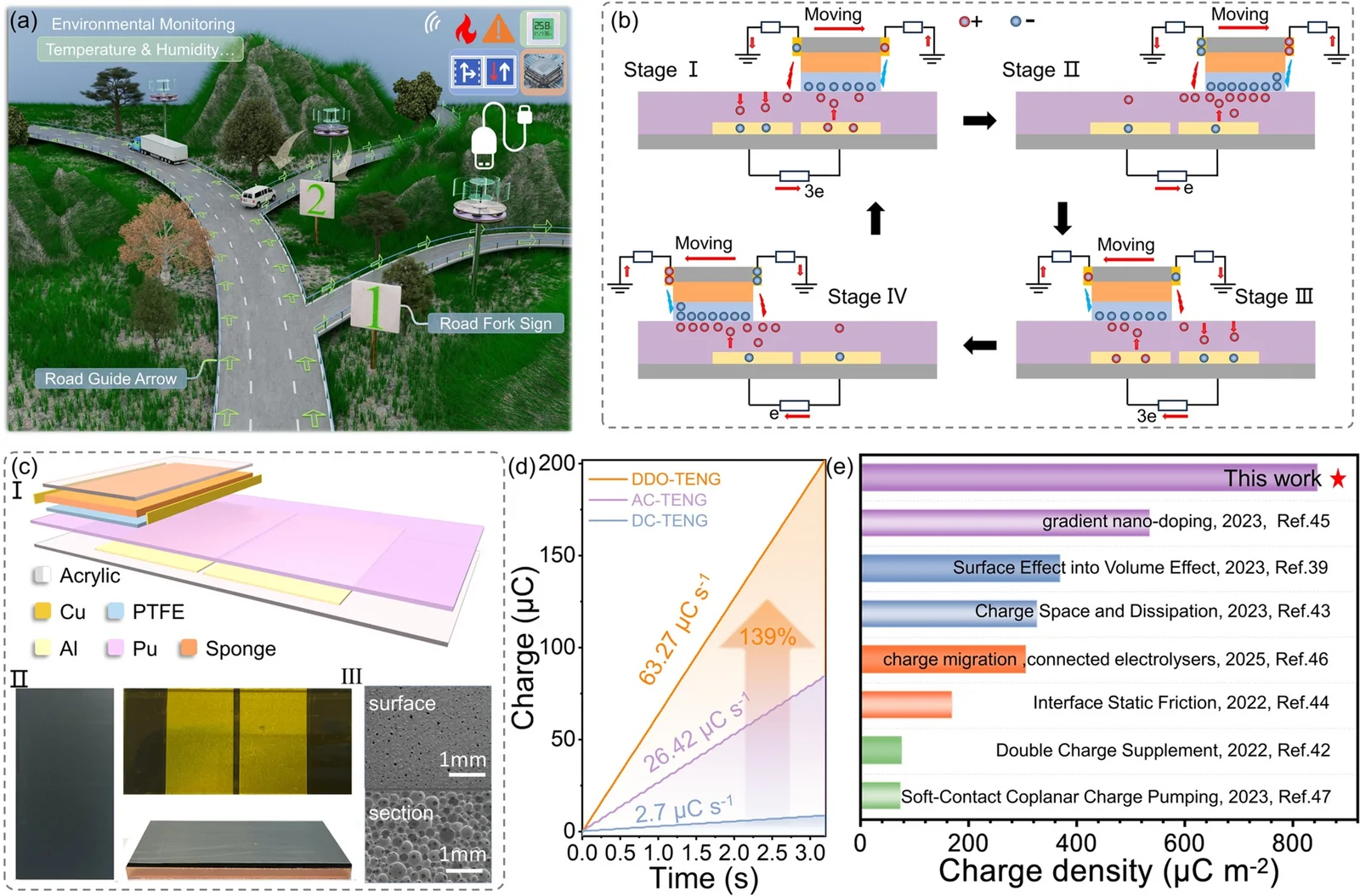
Working mechanism and electrical performance of DDO-TENG. **a** A scene diagram showing that TENG converts wind energy into electrical energy for highway signs and road markers. **b** Working principle of charge transfer in TENG. **c** 3D structure and Optical photographs of the horizontal sliding-mode DDO-TENG, and SEM of PU foam. **d** Charge output rate of DDO-TENG, AC-TENG and DC-TENG at 1 Hz. **e** A comparison of the charge density of DDO-TENG with that of typical TENG

In response to the challenging issue of power supply for infrastructure in wild and outdoor areas, the adoption of environmental mechanical energy conversion technologies, such as the capture of wind energy and pressure energy, can break through the dependence on traditional power grids, it is economy and environmentally friendly. TENG as an effective energy conversion technology is undoubtedly a good choice. Herein, DDO-TENG is installed on road sides to harvest wind energy or water energy in the environment and power road signs as shown in Fig. 1a, reducing the cost of road circuit construction and maintenance.

When PU foam is used as the dielectric layer, the numerous interconnected micron-scale pores inside it provide three-dimensional migration channels for charges. When the slider is in frictional contact with the stator, the bound charges generated by triboelectrification are not only distributed on the surface of the PU, but also migrate over short distances in a volume-wise manner toward the interior of the material along the pore channels under the drive of the contact electric field. Based on the migration behavior of charges within the dielectric material in the volume effect as well as the transfer characteristics of charges that air breakdown to form conductive channels in the electrostatic air breakdown effect, this work focuses on the influence mechanism of the coupled action of these two effects on the charge transfer process, and designs and constructs a DDO-TENG with a dual output mode. The working principle of TENG in one cycle as shown in Figs. 1b and S1. Initially, when the slider contacts the stator, due to triboelectric charging, there are equal amounts of positive and negative charges on the surfaces of PU and PTFE as shown in Fig. S1**a**. When the slider moves to the right to stage Ⅰ, the slider coincides with the bottom electrode (BE) of stator, there is a strong potential difference between the surface of the dielectric material and the electrode, and a strong charge leakage effect (charge migration) occurs inside the PU material. Therefore, due to electrostatic induction, a large number of transferred charges will be generated in the external circuit. Since the charges on the dielectric layer have not reached saturation, the electric field strength in the air gap between the side electrode on the slider and dielectric material can just ionize a small number of air molecules, and generate low corona current in the electrodes on both sides. If no side electrodes are there, charges will dissipate into the air as shown in Fig. S1b. Then, slider begin entering the charge space accumulation area (stage Ⅱ), the triboelectrification of tribolayers follows charge conservation law. When the charge density on dielectric material reaches saturation, according to Paschen's Law, the dielectric strength of air is approximately 3 kV mm^−1^; as long as the intensity of this electrostatic field exceeds the dielectric strength of the air between the two electrodes, the surrounding air will undergo partial ionization and start to conduct electricity. This causes electrons to flow from the dielectric material film to the collection electrode, thereby reducing the potential difference between the two electrodes and forming a conductive channel. And the charge flows into the side electrode through an external circuit, forming a positive current output in one side electrode and a negative current output in the electrode on the other side. Stage Ⅲ and Stage Ⅳ are similar to Stage Ⅰ and Stage Ⅱ, just that the direction of the charge flow will be opposite.

The three-dimensional (3D) structure and photographs of DDO-TENG are shown in Fig. 1c. Different from traditional TENGs, DDO-TENG is composed of a stator with volume effect and a slider which triggers the electrostatic air breakdown effect. Both the stator and the slider are integrated with electrode structures of specific functions. The aluminum film electrode on the bottom of the stator serves as an electrostatic induction electrode which can generate an alteranting current (AC) output. The side of the slider is equipped with a suspended copper (Cu) electrode, which acts as a charge capture electrode (CCE) for electrostatic air breakdown to achieve the output of a direct current (DC) signal. The tribolayer of slider is PTFE with a 1 mm back foam layer to reduce air voids and to ensure a better contact status. The stator uses an acrylic plate as the base, with an aluminum film covering its surface as the induction electrode, and a PU material with volume effect is used as the dielectric layer. Figure 1c(Ⅲ) shows the scanning electron microscope (SEM) image of PU foam. Inner of PU foam has many small holes, allowing soft contact during DDO-TENG operation, reducing wear and enhance the capability of charge migration. The inner porous network structure of the PU foam film to make sure that charges can easily hop in the surface state of the network.

Based on the coupling effect of triboelectrification and electrostatic induction triggered by the volume effect of the stator, as well as the electrostatic breakdown of the CCE in the slider, DDO-TENG can simultaneously generate alternating current and direct current outputs. And the output charge of AC and DC of the DDO-TENG reach 780 and 500 nC, respectively. In addition, the CCE exhibits the output characteristics of bipolar DC signals in single working cycle (Fig. S2). Through the designed DC collection electrodes and with the aid of the coupling effect between the volume effect and air breakdown, the output performance has achieved a 119% improvement compared to traditional AC-TENG, as specifically shown in Fig. S3. Moreover, the charge transferring rate achieves 139% improvement after optimization compared with single mode TENG (Fig. 1d). Hence, the energy conversion efficiency of DDO-TENG is further improved, and the charge density reaches 847.6 μC m^−2^. The total output of the DDO-TENG has been significantly improved compared with the previous works which force on optimizing the charge density and durability (Fig. 1e).

### Optimizing the Output Performance

The output performance of the DDO-TENG is influenced synergistically by multiple factors. Among them, the properties of the dielectric materials, such as material type, thickness, and friction coefficient, and the structural parameters of the device including electrode gap, electrode width, sliding distance, and motion speed, play a crucial role in the charge transfer efficiency and electrical energy output. Therefore, it is of great significance to improve output performance through the optimization of materials and structure. In this study, the influence mechanisms of the above-mentioned parameters on the current output characteristics and charge transfer process of the DDO-TENG are systematically investigated.

**Fig. 2:**
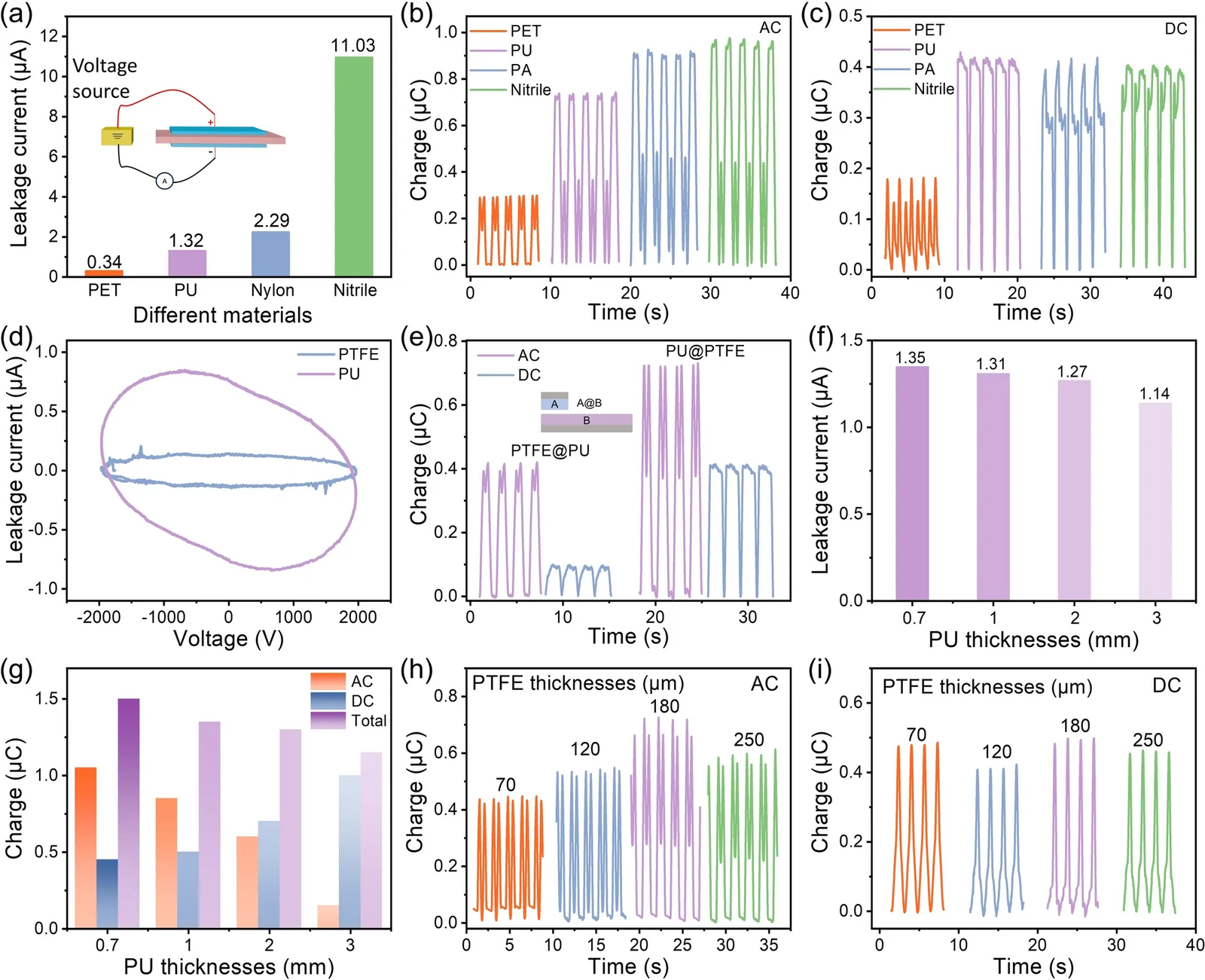
Performance optimization of dielectric materials in the triboelectric layer. **a** Leakage currents of different dielectric materials. **b** AC transferred charges of different dielectric materials. **c** DC transferred charges of different dielectric materials. **d** I-V curves of PU and PTFE. **e** Comparison of the output charges of TENG when PTFE and PU materials are used as the stator triboelectric layer. **f** Leakage currents of PU with different thicknesses. **g** AC and DC transferred charges of PU with different thicknesses. **h** AC charge outputs of PTFE with different thicknesses. **i** DC charge outputs of PTFE with different thicknesses

Subsequently, the influence of various parameters of dielectric materials on the output performance of DDO-TENG is studied. In order to screen out high quality materials suitable for the dielectric layer of the DDO-TENG, this study fixed the material of the slider dielectric layer as PTFE and first explored different positive dielectric materials for the stator. A 1 mm thick PU, a 50 μm PET, a 25 μm nylon, and a 20 μm nitrile are selected for the leakage current test. The results are shown in Fig. 2a, and the detailed current–voltage curves are shown in Fig. S4. Given the significant differences in the thicknesses of the dielectric materials, the thicknesses of the nitrile and nylon are in the micrometer range, the test results clearly show that the leakage current of the nitrile is the most significant, followed by that of the nylon, and the leakage intensities of both are significantly higher than that of the 1 mm PU foam. To verify the volume effect theory (the larger the leakage current, the greater the output), The charge transfer amounts and short circuit currents of the DC output and AC output of the DDO-TENG are tested, respectively, when four different materials are used as the dielectric materials. The relevant results are presented in Figs. 2b, c and S5, respectively. In the AC output, materials with larger leakage currents indeed exhibit better output performance. However, in the DC output, unexpectedly, the stronger the leakage current, the lower the output. Through analysis, the occurrence of this phenomenon is directly related to the charge behavior of triboelectric charging during the operation of DDO-TENG. In materials with stronger leakage currents, charges are more likely to migrate to the bottom, resulting in a significant reduction in the remaining charge amount on the surface of the material. Consequently, it is difficult to accumulate the charge threshold required for air breakdown with the side electrodes, ultimately leading to a decrease in the DC output. In addition, during the working process, it was observed that nitrile and nylon (PA) need closer contact to achieve a larger output.

Based on this phenomenon, we measured the friction coefficients of the four materials (Fig. S6a). Since the output of PET is significantly lower than that of the other three materials, we mainly explore PU, nitrile, and nylon. As the friction force test shown in that the friction coefficient of the nitrile is much higher than those of the PU and nylon, while the friction coefficients of the latter two are basically similar. Further analysis by combining the force applied to the materials and the frictional coefficient reveals that although the initial outputs of the nitrile and nylon are higher than that of the PU foam, their thin thicknesses in the micrometer range make them rely on higher contact pressures and friction coefficients, which directly leads to increased wear of the materials during the operation of DDO-TENG. Once the material has structural damage, the output of the device will drop sharply. In contrast, the structural advantages brought by the millimeter scale thickness of the PU material form a sharp contrast. This material can not only maintain a stable interfacial contact state with its sufficient thickness, but also keep its material structural properties stable even if a certain degree of wear occurs, thus ensuring that the output performance is not significantly affected. The comprehensive advantages brought by this thickness provide a reliable support for the long-term stable operation of TENG (the subsequent durability tests will further confirm this conclusion). In conclusion, considering the output performance, material durability, and the requirements of the actual application scenarios comprehensively, this work finally selected PU as the stator dielectric material for the subsequent study.

To further explore the influence of the volume effect on the DDO-TENG performance, a comparative leakage current test for PU and PTFE. As shown in Fig. 2d, when the scanning voltage increased from 0 to 2 kV, the leakage current of the PU foam reached maximum 840 nA, which is approximately 28 times that of the PTFE film (30 nA). This remarkable difference can be attributed to the unique porous structural characteristics of the PU material. Compared with the densely structured PTFE film, the porous structure of the PU material endows it with excellent leakage current conduction ability. This essential difference in structure leads to a distinct divergence in the electrical performance between the two. The porous structure of the PU effectively promotes the migration and diffusion of charges, thereby significantly enhancing its leakage current capacity. Based on the above test results, an exchange experiment of material configurations is further designed (Fig. 2e). The structure with PTFE as the slider tribolayer and PU as the stator tribolayers is denoted as PTFE@PU. Through tests of charge transfer, the output performance of the PTFE@PU structure is significantly better than that of the PU@PTFE in both AC and DC modes. This result indicates that the charge migration of the PU foam is significantly better than that of the PTFE film, which is consistent with the test output of the TENG and meets the proposed volume effect, that is, the higher charge migration in the thick dielectric film leads to better output performance.

In order to verify the influence of the leakage current capabilities of the same dielectric material with different thicknesses on the output performance. The leakage current of PU with different thicknesses as stator tribolayer are tested in Fig. 2f, and the same scanning voltage is increased from 0 to 2 kV to illustrate that the leakage characteristics of the same dielectric material will weaken with thickening. Then the charge transfer and current output of PU of different thicknesses are tested (Fig. 2g). The leakage current capability of the materials decreased, the total output performance of the DDO-TENG decreased, accompanied by an increase in material thickness. Due to the increase in the thickness of the dielectric material, the leakage capability of the material becomes weaker, the downward migration of the charge is blocked, and the AC output begins to decrease. However, due to triboelectrification, charges are constantly being generated here between the two dielectric materials. When the charge is not easy to migrate downward, the surface charge density increases, the electric field intensity of the air gap between the CCE and PU will increase rapidly. Hence, the air breakdown threshold is easier to reach and conductive channels formation, generating more DC output. The charge transfer is the largest when the PU thickness is 0.7 mm, and the capability to transfer the charge is directly proportional to the leakage capacity of the material, which satisfies the proposed volume effect. That is, the higher the charge migration capability in the thick dielectric film, the better the output performance. The friction coefficient of PU foams with different thicknesses shows that the friction coefficient is too large when the PU thickness is 0.7 mm (Fig. S6b). Hence, the dielectric material will be consumed too quickly during working process, which is not conducive to the long-term work of TENG, therefore, select 1 mm thick PU for the subsequent study.

After optimizing the dielectric material of the positive electrode of the stator, the influence of the negative dielectric material of the slider on the output performance of DDO-TENG is further studied. For the negative dielectric material, since PTFE has a low coefficient of friction and strong triboelectric performance, it is selected as the fixed negative material in this work. However, the influence of PTFE with different thicknesses on the AC and DC outputs still need be studied. The charge transfer is shown in Fig. 2h, i and the characteristics of the output current are shown in Fig. S7. It can be observed that the thickness of PTFE does affect the output performance of TENG. The reasons for this phenomenon are as follows: When the material is too thin, PTFE lacks sufficient rigidity and has limited deformability. During contact with the friction layer, issues such as local suspension and uneven fitting tend to occur, which leads to a significant reduction in the effective frictional contact area compared to the optimal thickness, as well as a substantial decrease in the total charge density generated by triboelectrification. When the material is too thick, PTFE exhibits reduced flexibility and excessive deformability. The frictional pressure cannot be evenly transmitted to the entire contact interface, easily forming a contact state of "local compaction and overall looseness". This results in a decrease in the effective frictional contact area compared to the optimal thickness, which in turn leads to a reduction in triboelectrification efficiency. Through optimizations on dielectric materials, 1 mm thick PU and 180 μm thick PTFE are selected as the dielectric materials for DDO-TENG. The AC output charge of the sliding TENG reaches 700 nC, the current output reaches 3.98 μA, and the DC reaches 450 nC, 2 μA, respectively.

### Optimizing the Structure and Durability

**Fig. 3:**
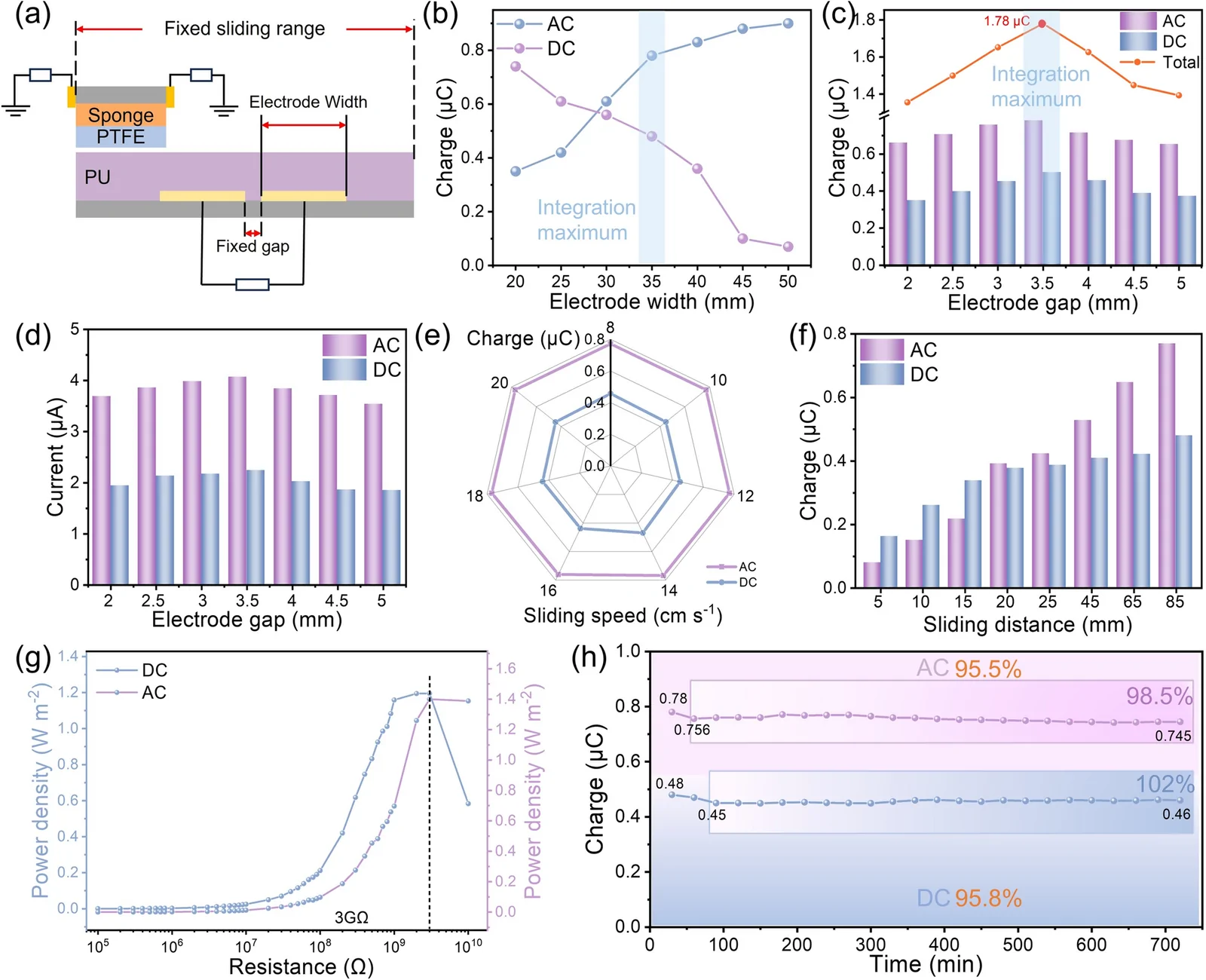
Optimization of the structural performance of DDO-TENG. **a** Schematic diagram of the planar structure of TENG. **b** Transferred charges with different widths of the stator electrodes. **c** Transferred charges with different gaps between the two electrodes. **d** Current output with different gaps between the two electrodes. **e** Transferred charges under different sliding speeds. **f** Transferred charges with different sliding distances. **g** Maximum output power of alternating current and direct current under different resistances ranging from 1 MΩ to 10 GΩ. **h** Transferred charges when TENG operates continuously for 12 h

Structural design of TENG also has an inseparable influence on the output performance and durability. Figure 3a is a planar schematic diagram of the structural parameters of the DDO-TENG, clearly presenting the core parameters, such as electrode width, electrode gap, sliding speed and sliding distance. Subsequent research will carry out optimization for the above-mentioned parameters to enhance the output performance of the device. Firstly, transferring charge was performed on different electrode widths as shown in Fig. 3b. By changing the electrode width with a gradient of 0.5 cm, it is found that the AC output of TENG is proportional to the electrode width, that is, the wider the electrode width, the greater the AC output. Meanwhile, the DC output is inversely proportional to the electrode width. Due to the shielding charge characteristics of the bottom electrode, the charge density of the dielectric layer above the BE is higher than that of the charge space accumulation area. When the slider slides over the BE, the charge on the lower surface of PTFE is shielded by the charge on the upper surface of PU, which leads to a decrease in the potential difference between the electrode on the slider and the PTFE. Hence making it difficult for electrostatic breakdown to occur, as indicated by the output generated at different stages during the operation cycle in the current output (Fig. S8). The results reveal significant electrostatic breakdown phenomena occurring in the sliding type TENG when the slider is positioned over the non-overlapping region of the BE. Meanwhile, localized discharge events are also observed during the overlapping phase with the BE. Part of the surface charge accumulated on the PU fails to migrate to the low surface, resulting in residual charge and dielectric breakdown between the side electrodes. The output performance of DDO-TENG requires the synergy of AC output and DC output to reach the optimum. When the electrode width is 3.5 cm, the transferred charges of the AC and DC are 0.78 and 0.47 μC, respectively. Due to the design of the DDO-TENG, the DC part will generate two outputs with different phases. The total transferred charge of DDO-TENG reaches a maximum value of 1.72 μC.

Subsequently, the influence of the gap of BE on its output performance is studied, the transferring charge and output current when the electrode gap increases from 2 to 5 mm are shown in Fig. 3c, d. When the gap increases from 2 to 3.5 mm, the transferred charges and short circuit current of both AC and DC output are increasing, the total transferred charge reached 1.78 μC. But when the gap gradually increases further, the output performance begins to show a downward trend. When the electrode gap is relatively small, the strong leakage characteristics exhibited by the PU foam will cause the lateral migrating of charges inside it. This lateral migrating phenomenon will interfere with the normal path of the charges and weakening the vertical migrating that the charge should originally have. As the electrode gap increases, the migrating of the charge shows obvious regular changes. Specifically, the lateral charge migrating lead to output decreasing, while, in contrast, the vertical migrating increasing. This can be attributed to the fact that the change in the electrode gap will adjust the electric field distribution, affecting the force on the charges in different directions. When the electrode gap increases to a large enough extent, the lateral charge migrating almost disappears, and a larger electrode gap will lead to a decrease in the electric field strength, resulting in a reduction in the number of induced charges.

It is found that the sliding speed does not affect the saturated charge, and the transferred charges of its AC output and DC output remain at approximately 0.78 μC and approximately 0.5 μC, respectively, when the sliding speed is between 8 and 20 cm s^−1^, as shown in Fig. 3e. As charge capture electrode, the characteristics of CCE are closely related to the DC output. It mainly captures charges through electrostatic breakdown and air breakdown, which means that it can only capture charges in regions where the surrounding charge density is high enough. The speed will only affect the rate of charge transfer, and will not affect the charge density increase. Regardless of the speed, when the surface charge density of the dielectric material saturated, the charge transferred on the BE and CCE in one working cycle will not change. But the current will increase with the increase in speed, detailed data are shown in Fig. S9. As the sliding distance gradually increases, the frictional area of the dielectric material correspondingly becomes larger, and more charges will be generated and the charge density increases. Meanwhile, the number of migrating charges inside the PU also increases accordingly. And the area where CCE captures charges will gradually increase, causing both the AC and DC output to show an increasing trend, as shown in Fig. 3f. The output power, current and voltage of the AC and DC of the DDO-TENG under different resistances ranging from 1 MΩ to 10 GΩ as shown in Figs. 3g and S10. The power density of AC/DC output channel reaches 1.43 and 1.19 W m^−2^, respectively. After the material and structural optimization of the DOM-TENG was completed, it has been confirmed that the synergistic effect of the volume effect and electrostatic breakdown significantly enhances the output of the TENG.

To further verify its output stability, a long-term operation test was conducted, as shown in Fig. 3h. During the continuous operation for 12 h, the AC and DC output exhibited slight attenuation within the first 60 and 100 min, respectively. However, after entering the stable operation stage, both outputs remained at a constant level. After 12 h of operation, the AC output reached 98.5% of the initial stable value, while the DC output showed a slight increase of 102%. Notably, throughout the entire operation cycle, both the AC and DC outputs consistently remained above 95% of their initial values. This excellent stability can be attributed to two factors. On the one hand, the surface charge density of the dielectric material gradually reached a saturated equilibrium state as the operation time increased, suppressing the performance fluctuations caused by initial charge dissipation. On the other hand, the flexible structural properties of PU enabled soft contact at the interface, effectively reducing the impact of mechanical wear on the output. The dynamic curves of transferred charges during the operation process shown in Fig. S11.

### Optimizing the Output Performance of Rotary Mode DDO-TENG

**Fig. 4:**
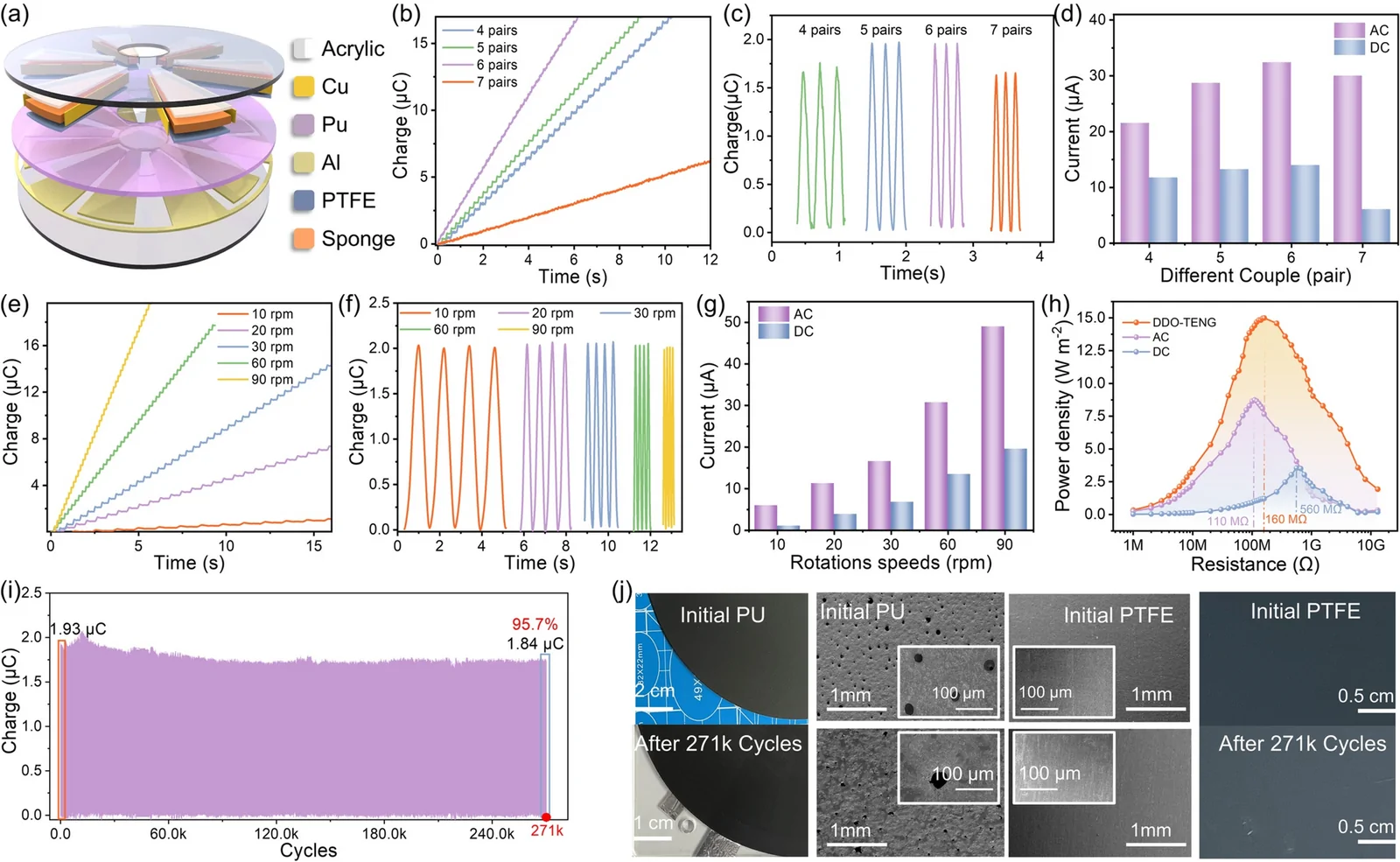
Output performance of the rotational DDO-TENG. **a** Schematic diagram of the 3D structure of the rotational DDO-TENG. **b**-**c** DC and AC transferred charges with different numbers of stator electrode pairs. **d** DC and AC current outputs with different stator electrode pairs. **e**–**f** DC and AC transferred charges at different rotation speeds. **g** DC and AC current outputs of different rotation speeds. **h** Maximum output power of AC and DC under different resistances ranging from 1 MΩ to 10 GΩ. **i** durability test of the transferred charge of DDO-TENG. **j** SEM images of PTFE film and PU foam surface at initial and after 271 k cycles of continuous testing

Due to the advantage that unidirectional rotational motion has in realizing continuous energy harvesting, based on the optimized parameters of the sliding DDO-TENG, and on the basis of fully considering its periodic working characteristics, the rotary DDO-TENG was designed through the rational layout of the sliding DDO-TENG, as shown in Fig. 4a**.** It is composed of a stator and a rotor. In the stator, radial electrodes are designed between acrylic base and PU. The rotor with the half number of CCE pairs, using PTFE as the tribolayer. The detailed dimensions and design details are presented in the experimental section. The photograph of the rotating DDO-TENG is shown in Fig. S12.

Firstly, the output charge and current of the 4–7 BE pairs of the rotary DDO-TENG are tested. When the number of electrode pairs is between 4 and 6, the transferred charges and current of DC and AC of rotary DDO-TENG all increase gradually. While when it exceeds 6 pairs, the output begins to decline, as shown in Fig. 4b, d. When the number of electrode pairs increasing, it can collect the charges fully, thereby increasing the output current and voltage. Each additional pair of electrodes is equivalent to adding a charge collection and output unit, and these units cooperate with each other to jointly improve the output performance. However, as the number of electrode pairs further increases, the distance between BE gradually decreases, and the electrostatic coupling effect begins to strengthen and lead to the redistribution of charges between the electrodes, causing some charges to be unable to be effectively output, and further reducing the output performance of DDO-TENG. When the number of electrode pairs is too much, this electrostatic coupling effect becomes extremely significant. In order to further determine whether a degradation in output performance occurs when the number of electrode pairs is increased too much, 12 electrode pairs are designed to verify this. Obviously, the output decline, as shown in Fig. S13. Hence, the output shows a trend of increasing first and then decreasing with the increase in the number of electrode pairs.

Subsequently, the influence of the rotational speed on the output of DDO-TENG is shown in Fig. 4e–g, the transferred charges and short circuit current of AC and DC are displayed, respectively. By analyzing the charge transfer and short circuit current of 6 pairs of electrodes at different rotational speeds, the AC output is stable at low-frequency rotational speeds. For DC output, the increase in rotational speed leads to a significant increase in the amount of frictional charge generated per unit time. High speed causes the potential difference in the gap between the side electrode and the PU material to show an accelerating upward trend, enabling the voltage to reach the air breakdown threshold more rapidly. With the continuous increase in rotational speed, the charge transfer rate exhibits an obvious characteristic of positive correlation growth, ultimately achieving an improvement in the DC output performance. In addition, after optimization, the power density of AC output part achieves 8.74 W m^−2^ with a matching resistance of 110 MΩ, the DC part is 3.54 W m^−2^ at 560 MΩ, and the DDO-TENG achieves up to15 W m^−2^ at 160 MΩ, 1.72 and 4.4 times than the AC and DC mode, respectively (Fig. 4h). The short circuit current and voltage of DDO-TENG under different resistances are shown in Fig. S14.

Subsequently, a durability test was carried out based on the above optimized structural parameters. The results show that after 271,800 working cycles, the transferred charge can still maintain 95.7% of the initial value (Fig. 4i). The comparison of the transferred charge amounts in the initial state and after the cycles is shown in detail in Fig. S15. The optical photographs and SEM images of the tribomaterials PU and PTFE in the initial state and after durability test are shown in Fig. 4j, and just slight wear marks on the surface of the materials are observed. However, the durability test show that such slight wear has no significant impact on the output performance of the device. The unique porous network structure of the PU material, with its low surface contact force and self-polishing effect, effectively improves the soft contact interface of DDO-TENG, thus significantly reducing the energy loss.

## Application Demonstrations

**Fig. 5:**
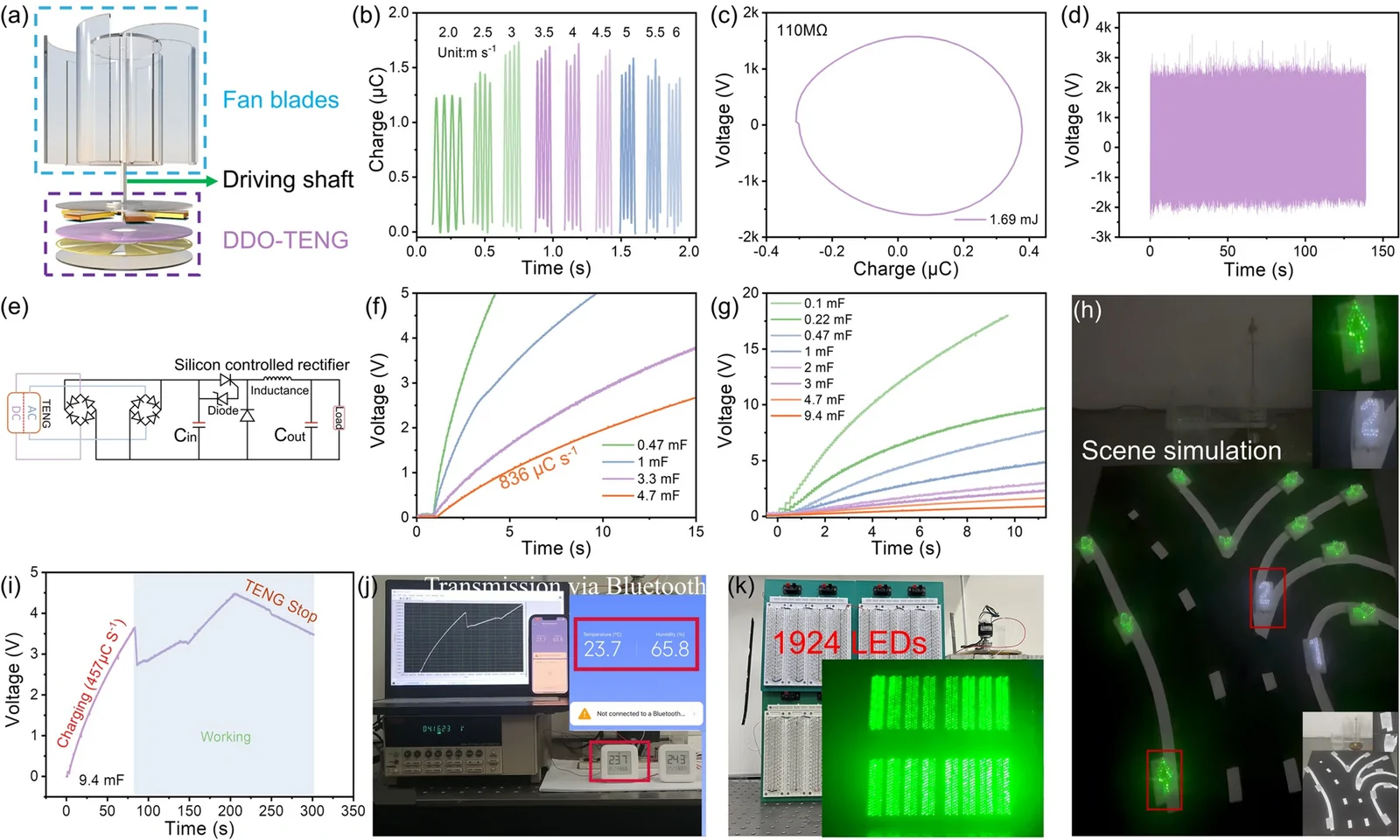
Application demonstrations of the rotational DDO-TENG. **a** 3D structure diagram of the rotational TENG with Fan blades. **b** Transferred charges at different wind speeds. **c** Voltage charge curve when the external load is 110 MΩ. **d** Voltage at a wind speed of 4 m s^−1^. **e** Power management circuit. **f** Charging different capacitors at a fixed rotation speed of 60 rpm. **g** Charging different capacitors at a fixed wind speed of 4 m s^−1^. **h** Road indicator lights and road signs composed of LED lights, which are powered and illuminated by the rotational TENG at a wind speed of 4 m s^−1^. **i** Voltage diagram of powering two parallel Bluetooth hygrometers by rotating DDO-TENG at a rotation speed of 60 rpm. **j** Physical diagram of powering two parallel Bluetooth hygrometers by rotating DDO-TENG at a rotation speed of 60 rpm. **k** Lighting 1924 green LEDs by rotating DDO-TENG at a rotation speed of 300 rpm

To demonstrate the practical application capabilities of the devices we have designed, wind power is adopted as the driving source here. A 3D structural diagram of the rotary DDO-TENG equipped with fan blades for harvesting wind energy is shown in Fig. 5a. The upper layer of the device is responsible for converting wind energy into rotating mechanical energy. The lower layer is a rotary DDO-TENG, which is used to convert mechanical energy into electrical energy. The upper and lower layers are mechanically connected through a drive shaft. Subsequently, the effect of different wind speeds on the output charge of wind-driven DDO-TENG are tested, as shown in Fig. 5b. The voltage-charge curve at an external load of 110 MΩ indicates that each working cycle of DDO-TENG can output 1.69 mJ of energy at a wind speed of 4 m s^−1^, as shown in Fig. 5c. The voltage of DDO-TENG with 6 BE pairs can reach 2.5 kV under the wind energy drive, as shown in Fig. 5d. Then, a power management circuit (PMC) is designed to couple the rectified AC and DC outputs, as shown in Fig. 5e. The optical photograph of the PMC is shown in Fig. S16. It is mainly composed of a full rectifier bridge, capacitors, diodes, triode and inductors. All components jointly process the output of DDO-TENG, providing a stable power supply, which effectively enhances the reliability and practicality of the power supply system. As shown in Fig. 5f, g, rotary DDO-TENG can effectively charge different capacitors at a wind speed of 4 m s^−1^ and a rotation speed of 60 rpm, respectively.

Subsequently, a scenario of road traffic lights powered by a rotating DDO-TENG under wind speed of 4 m s^−1^ is simulated, as shown in Fig. 5h and Video S1. DDO-TENG can harvest random wind energy in the outdoor environment and supply energy for road signs and fork indication signs, improving vehicle driving safety. Additionally, two Bluetooth hygrothermographs are powered continuously by DDO-TENG though charge a 10 mF capacitor at 1 Hz with the charging rate of 457 μC s^−1^, and the hygrothermographs could work continuously over 3 min, as shown in Fig. 5i, j and Video S2. Finally, the rotary DDO-TENG is able to light up 1924 LEDs simultaneously at 300 rpm, as demonstrated in Video S3. These practical application demonstrations prove that DDO-TENG can efficiently convert mechanical energy into electrical energy, providing better technical support for the power supply of outdoor devices.

## Conclusion

In this study, a DDO-TENG with dual output mode based on volume effect and electrostatic breakdown is proposed. Through the optimal of dielectric materials, the AC output is achieved based on the charge migration within the materials. Meanwhile, the electrostatic breakdown is triggered, generating a DC output. Through the synergistic effect of the above mechanisms, the coupling effect of the AC and DC outputs is successfully realized. Which effectively improves the charge density of DDO-TENG and can converts the wind or water energy in the surroundings into electrical energy efficiently. Compared with a single, conventional TENG, the output of DDO-TENG with the synergistic effect has been significantly enhanced. The output power density reached 15 W m^−2^, and the charge density reached 847.6 μC m^−2^. Moreover, the durability tests on DDO-TENGs show that the output performances can still be maintained at over 95% of the initial after long time operation. Effectively ensuring its strong stability working mode in practical applications. The practical application of DDO-TENG can light up 1,924 LEDs and two parallel Bluetooth hygrothermographs simultaneously, verifying the practicality and effectiveness of DDO-TENG. Moreover, it can continuously power electronic devices under the wind speed ranging from 2 to 10 m s^−1^. Broadened the solution strategy for strong durability and high output performance of TENG.

## Supplementary Information

Below is the link to the electronic supplementary material.

Supplementary file1 (DOCX 8494 KB)

Supplementary file2 (MP4 3632 KB)

Supplementary file3 (MP4 10624 KB)

Supplementary file4 (MP4 6807 KB)

## Data Availability

Data are available from corresponding authors upon reasonable request.
