# Supplementary material for: High Durability Sliding TENG with Enhanced Output Achieved by Capturing Multiple Region Charges for Harvesting Wind Energy
Source: Nanomicro Lett. 2026 Jan 7;18:199. doi: 10.1007/s40820-025-02043-1 (PMC12779796; doi:10.1007/s40820-025-02043-1)
Supplement: Supplementary file 1 — Supplementary file1 (DOCX 8494 KB) [file 40820_2025_2043_MOESM1_ESM.docx]

Supplementary Information for

**High Durability Sliding TENG with Enhanced Output Achieved by Capturing Multiple Region Charges for Harvesting Wind Energy**

Wencong He^1, *^, Yunchuan Liu^1^, Junhao Jin^1^, Jiahao Cai^1^, Buyong Wan^1^, Jie Chen^1^, Xiaohong Yang^1, *^ and Chenguo Hu^2, *^

^1^ College of Physics and Electronic Engineering, Chongqing Normal University, Chongqing 401331, P. R. China

^2^ School of Physics, Chongqing Key Laboratory of Soft Condensed Matter Physics and Smart Materials, Chongqing University, Chongqing 400044, P. R. China

* Corresponding authors. E-mail: [hewc@cqnu.edu.cn](mailto:hewc@cqnu.edu.cn) (Wencong He); [xiaohongyang@cqnu.edu.cn](mailto:xiaohongyang@cqnu.edu.cn) (Xiaohong Yang); [hucg@cqu.edu.cn](mailto:hucg@cqu.edu.cn) (Chenguo Hu)

**Supplementary Figures**


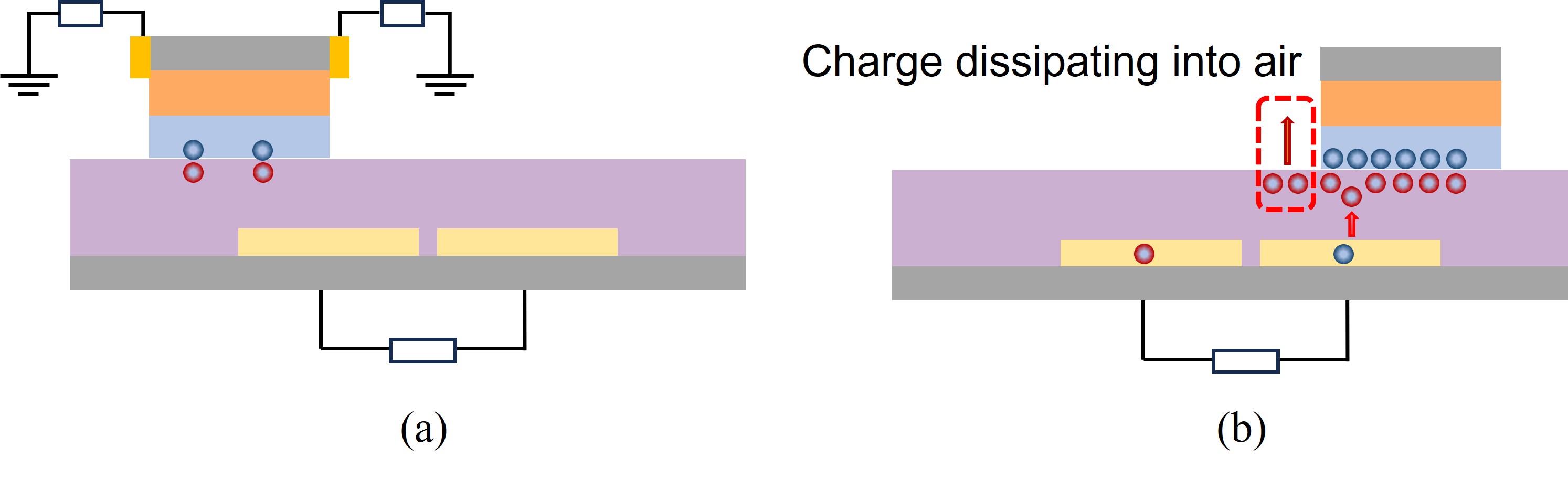


# **Fig.** **S1** Charge Dissipation Principle of TENG. **a** Initial work phase of TENG. **b** TENG charge dissipation operation without side electrodes


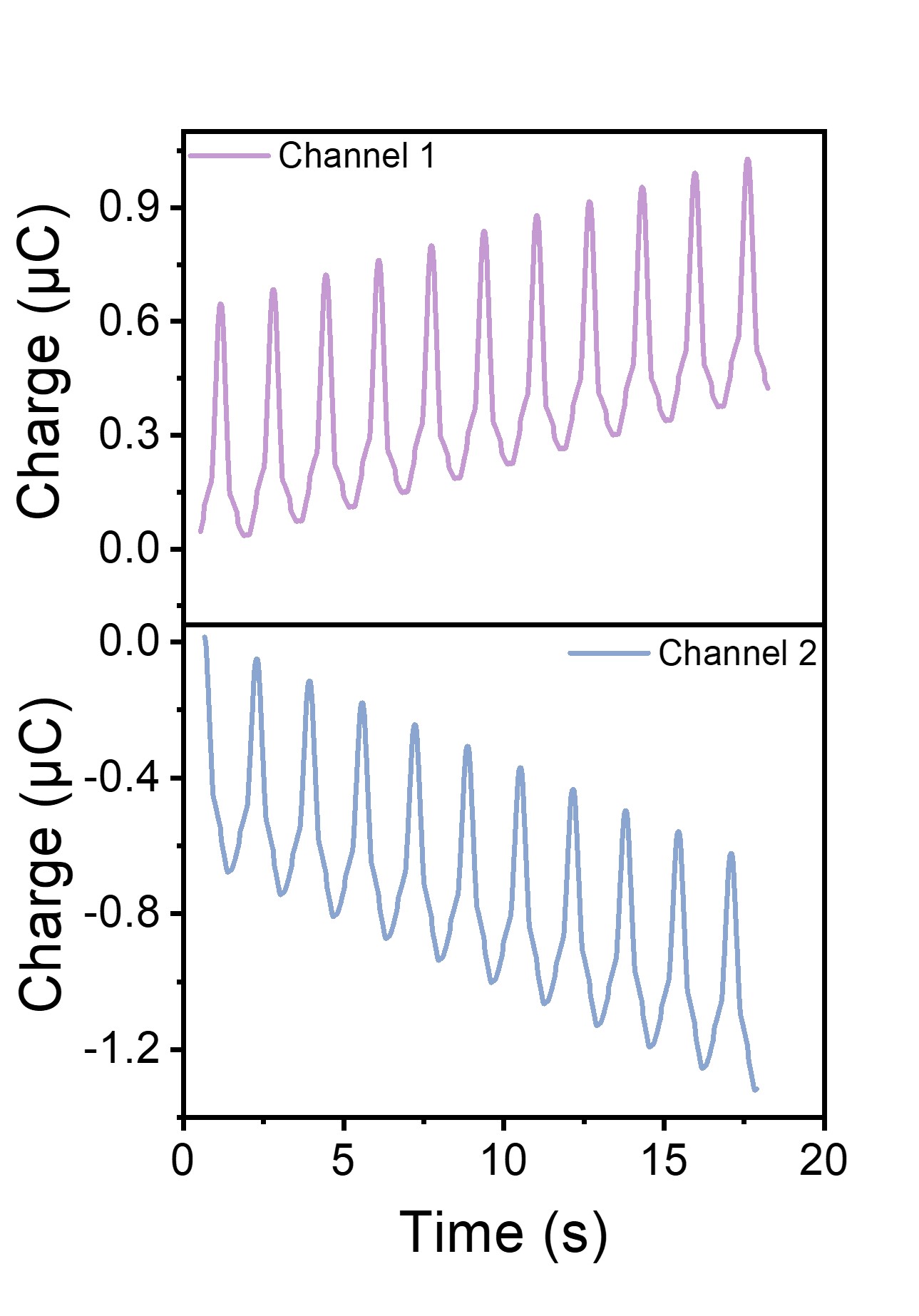


# **Fig.** **S2** Dual channel transfer charge of DC TENG


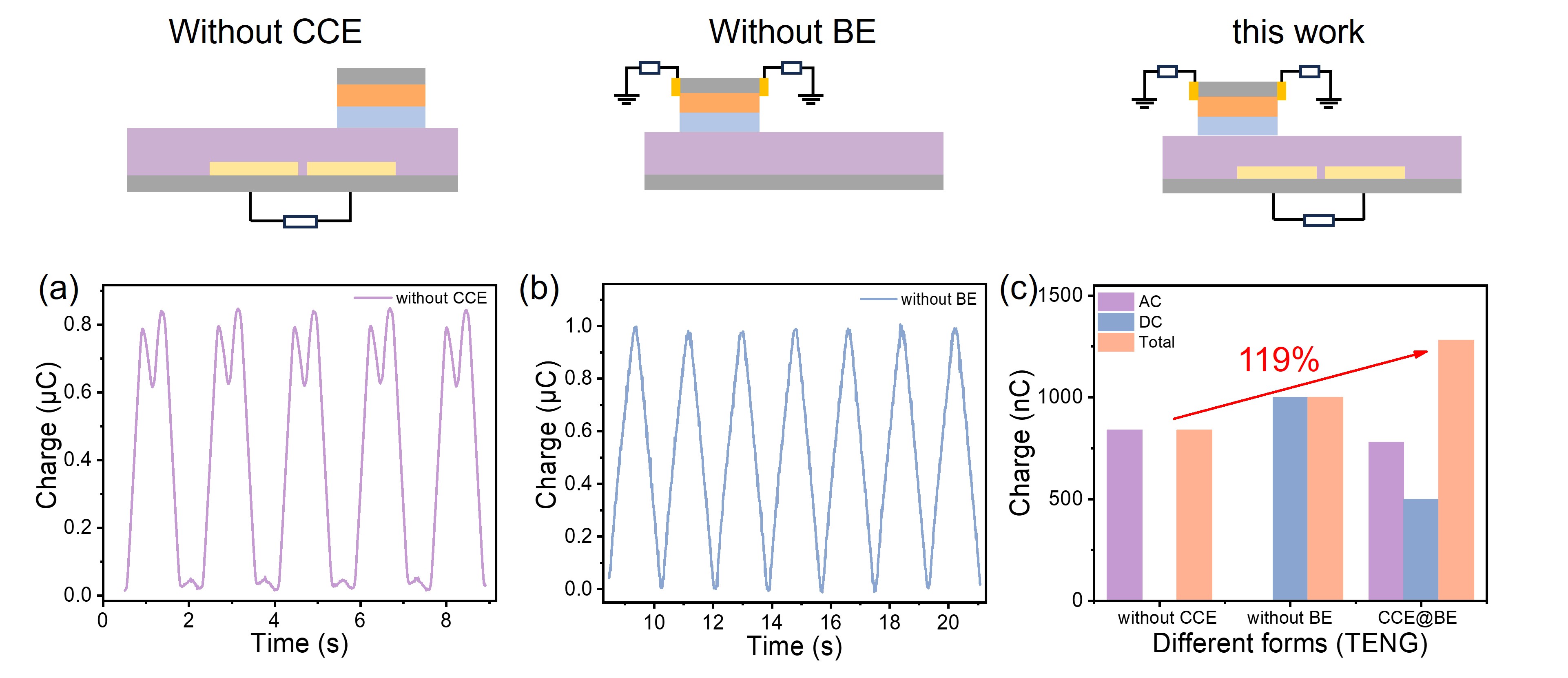


# **Fig.** **S3** Charge transfer in different types of TENGs. **a** Charge transfer without a collection electrode on the slider. **b** Charge transfer without a bottom electrode on the stator. **c** Comparison of charge transfer with both a collection electrode and a bottom electrode


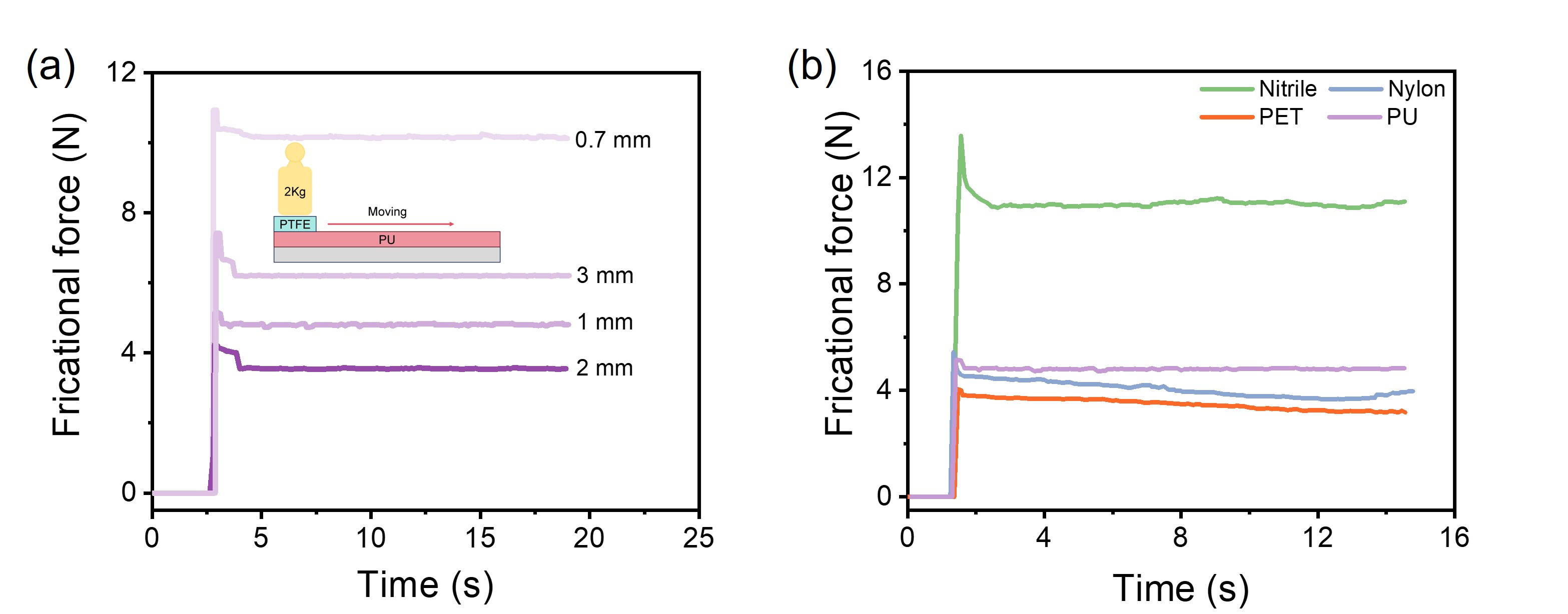


# **Fig.** **S4** The friction coefficient of the materials. **a** The friction coefficients of different PUs. **b** The friction coefficients of different stator dielectric materials


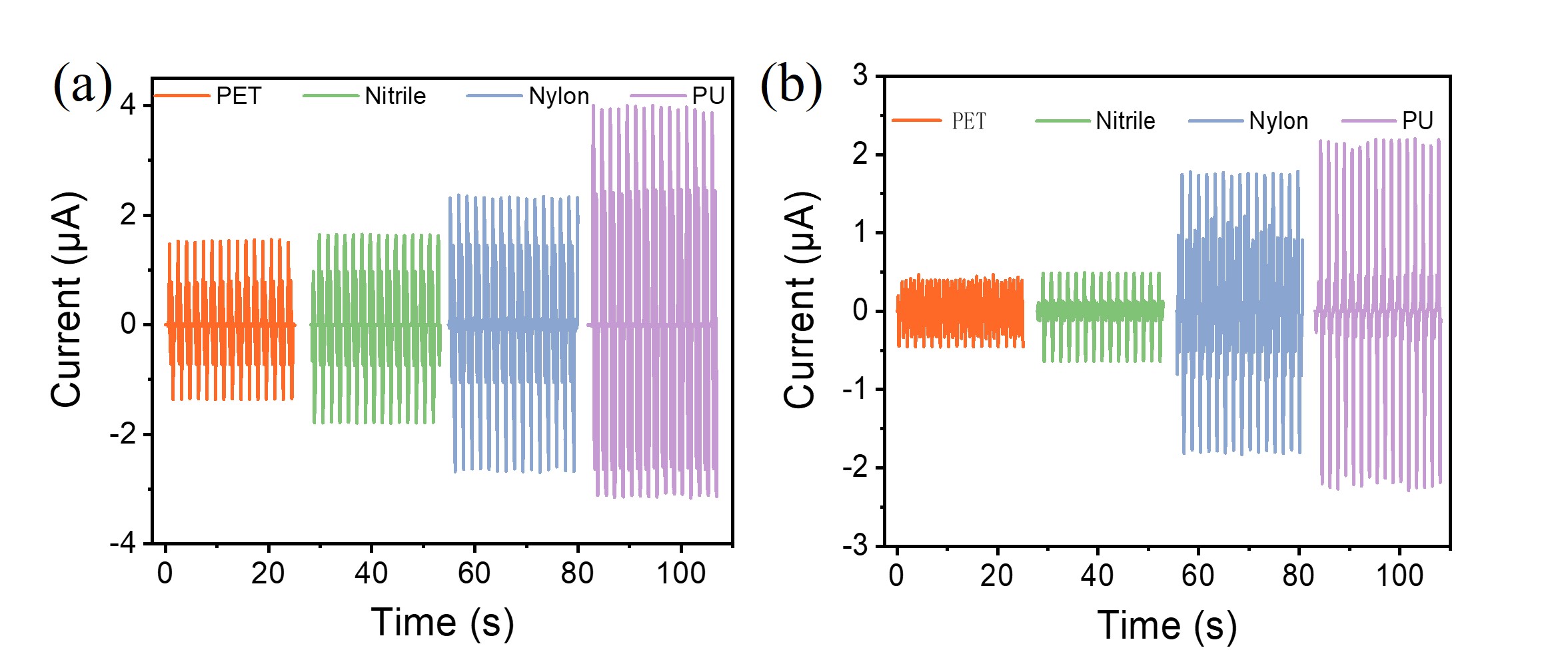


# **Fig.** **S5** Current output for different dielectric materials of stator. **a** AC currents of different positive dielectric materials. **b** DC currents of different positive dielectric materials

#
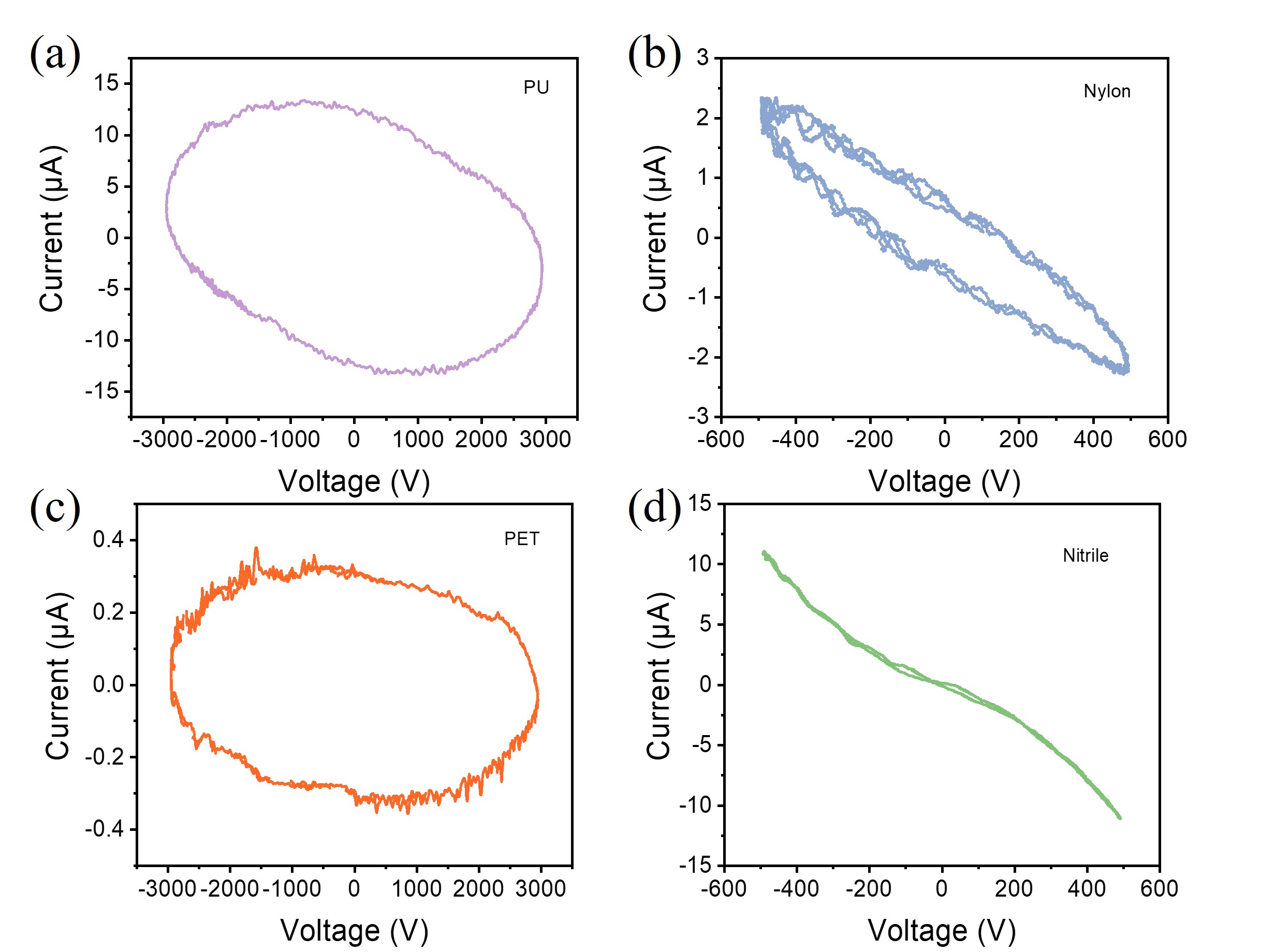


# **Fig.** **S6** I-V curves of different positive materials

#
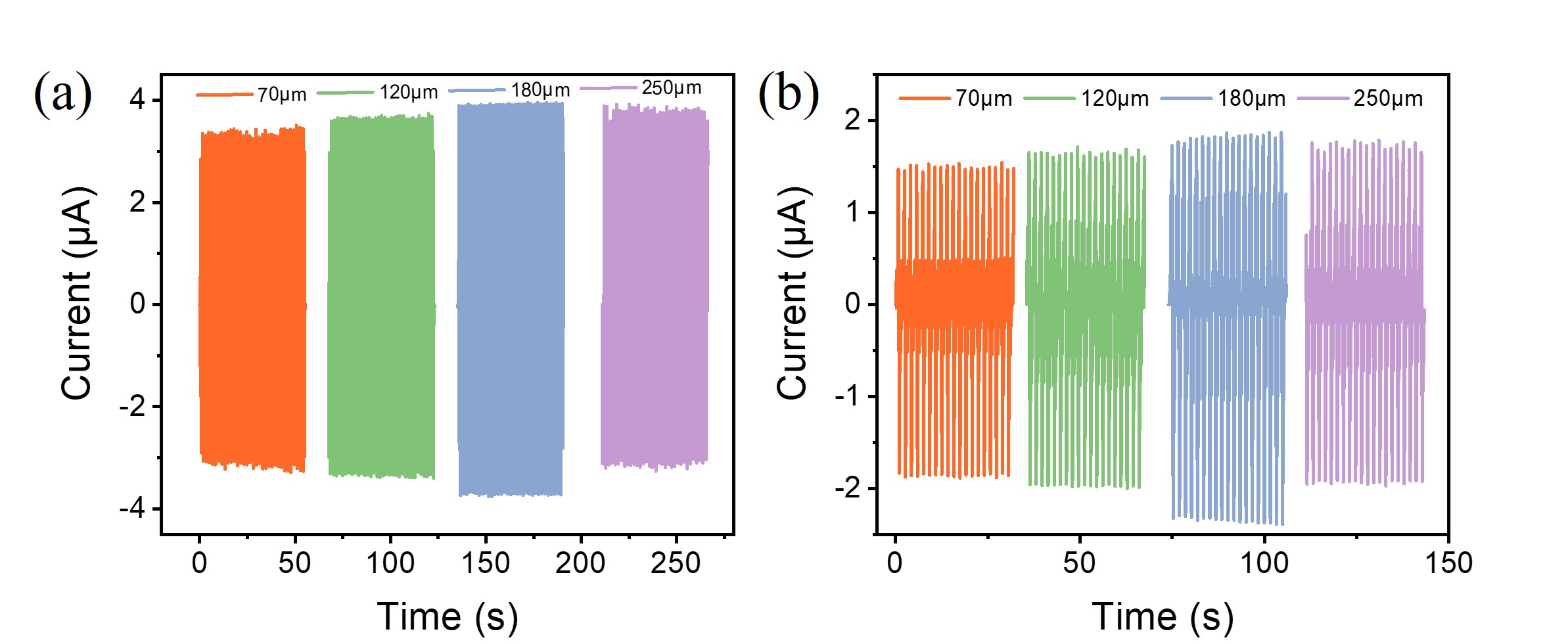


# **Fig.** **S7** Current output for different thicknesses of PTFE. **a** AC current output for PTFE of different thicknesses. **d** DC current output for PTFE of different thicknesses


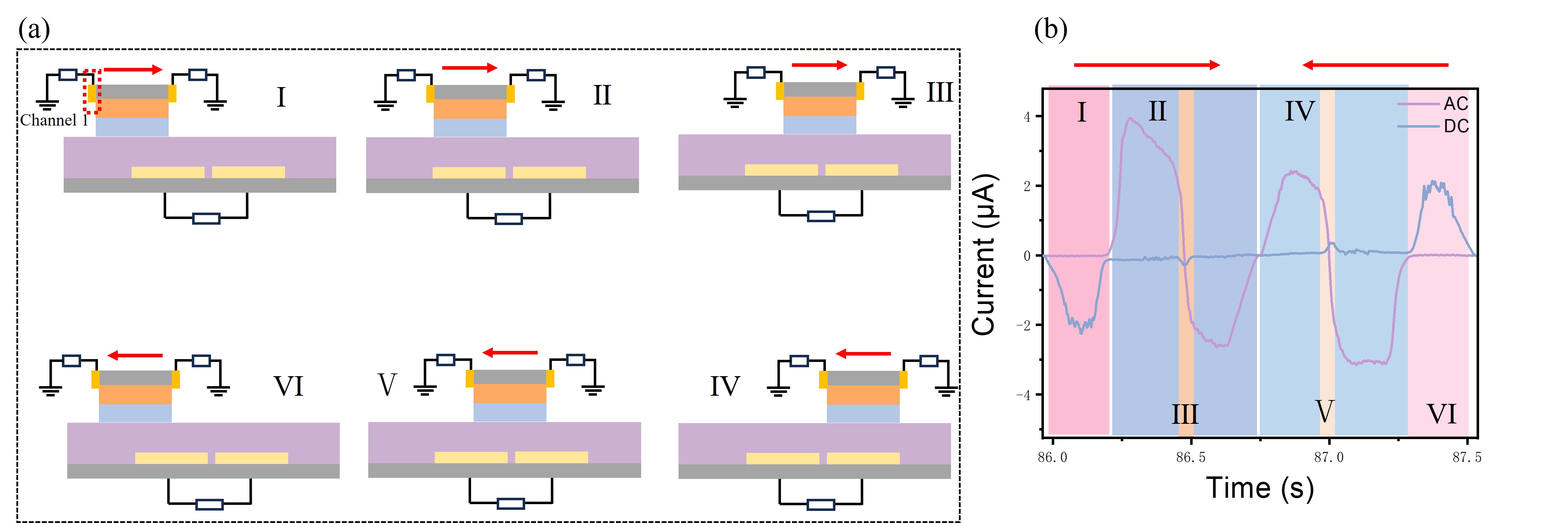


# **Fig.** **S8** Current output characteristics. **a** Different stages of work. **b** AC and DC short-circuit current output generated by the Channel 1 electrode and the bottom electrode of the slider at different positions

#
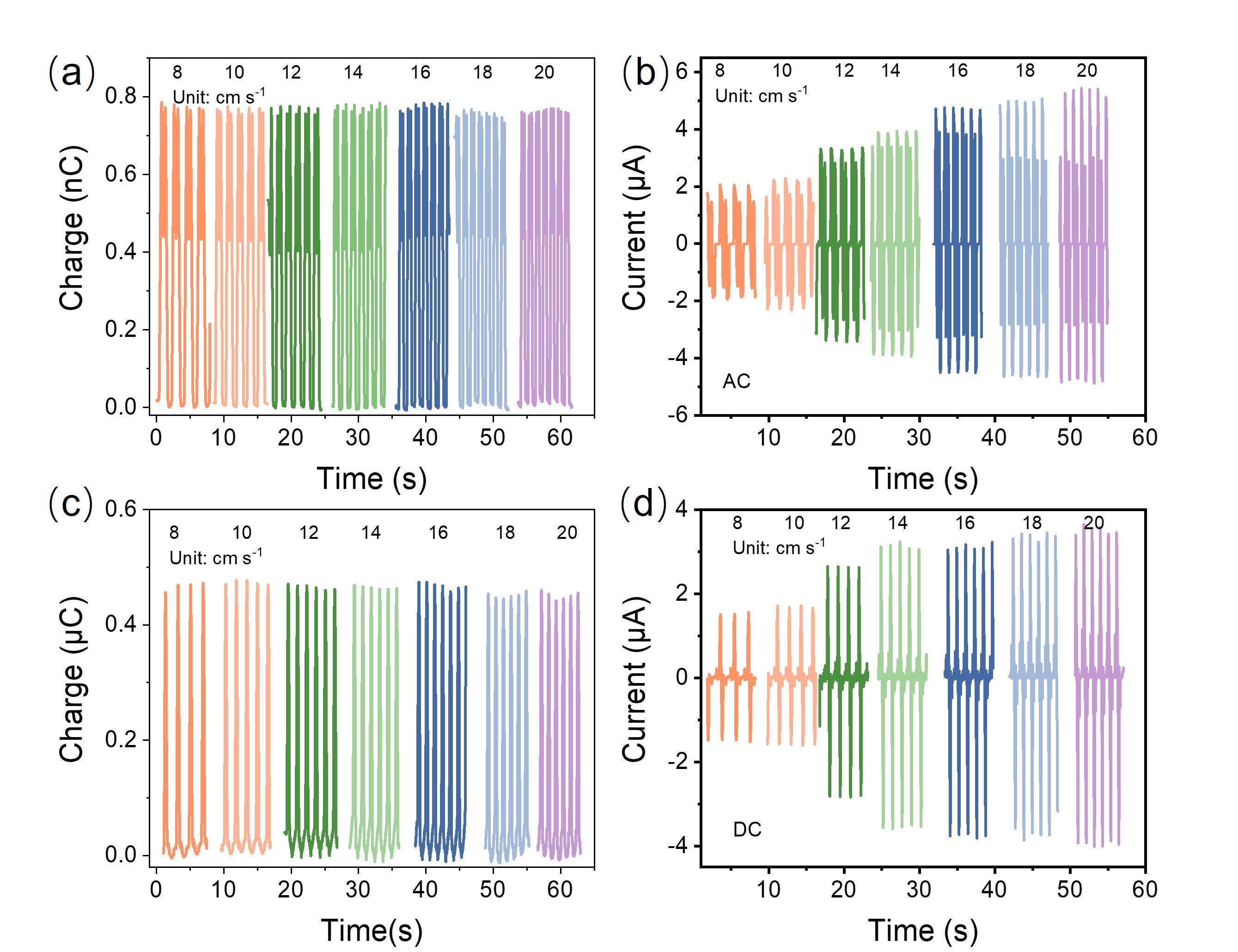


# **Fig.** **S9** The short-circuit current and the transferred charge of the sliding-type TENG under different speeds. a, **b** Transfer charge and short-circuit currents in AC. c, **d** Transfer charge and short-circuit currents in DC

#
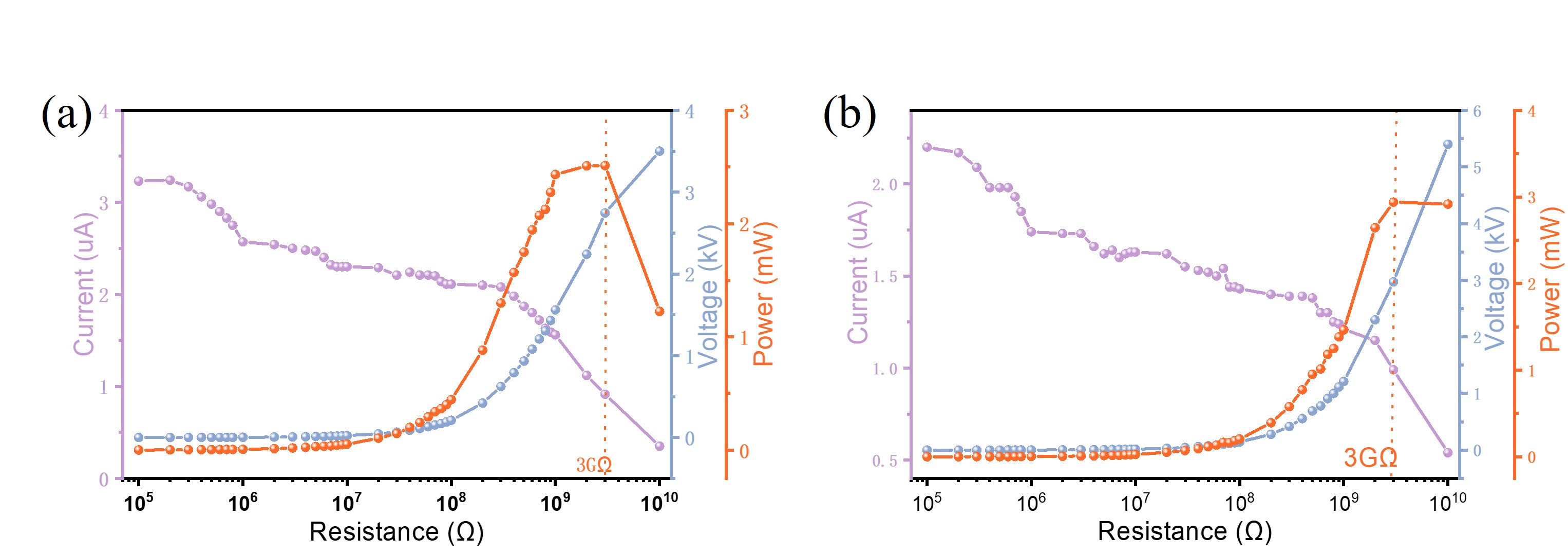
**Fig.** **S10** The short-circuit current and voltage of the sliding-type TENG under different resistances


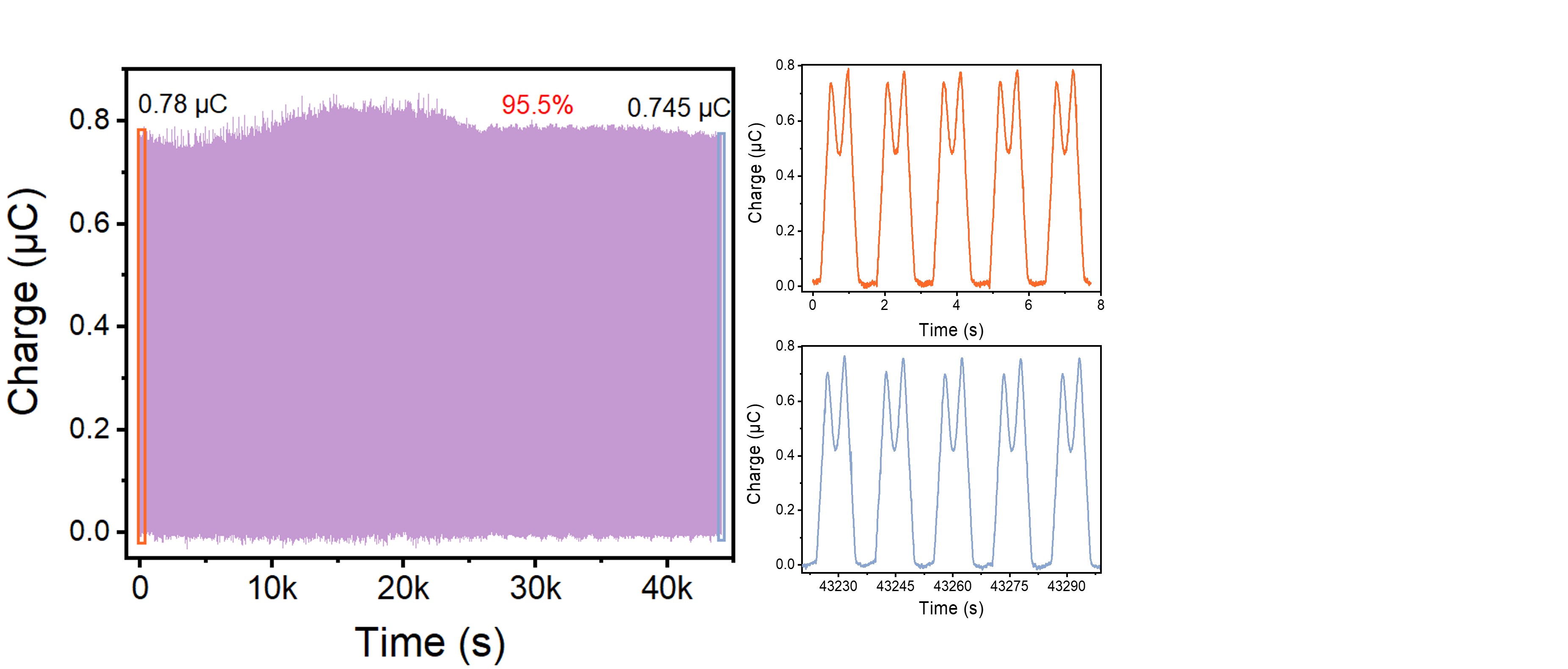


# **Fig. S11** Durability test of the sliding-type DDO-TENG, charge transfer from the beginning to after 12 hours


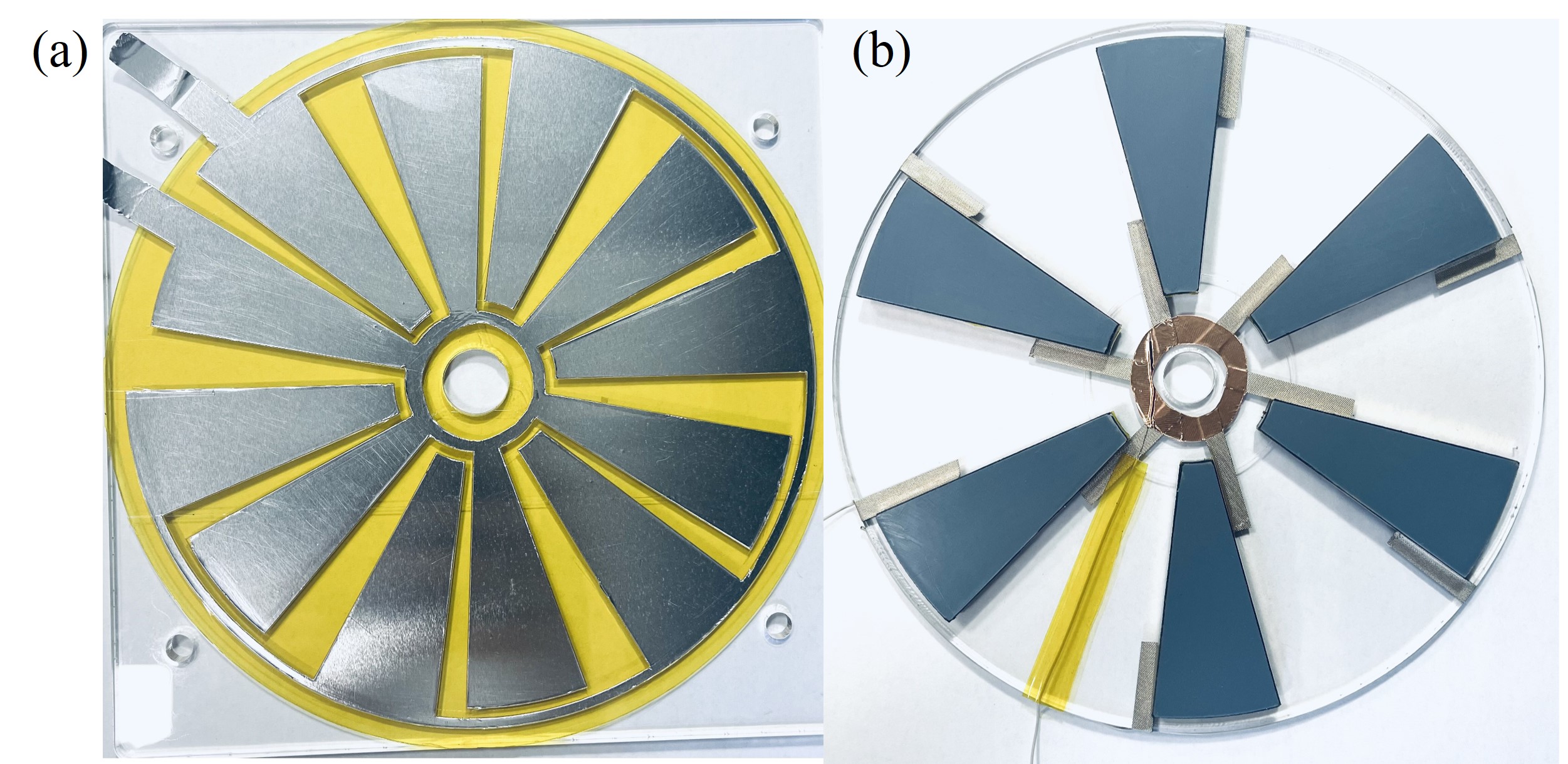


**Fig.** **S12** Optical photos of the rotating type DDO-TENG. **a** Schematic diagram of the stator. **b** Schematic diagram of the rotor


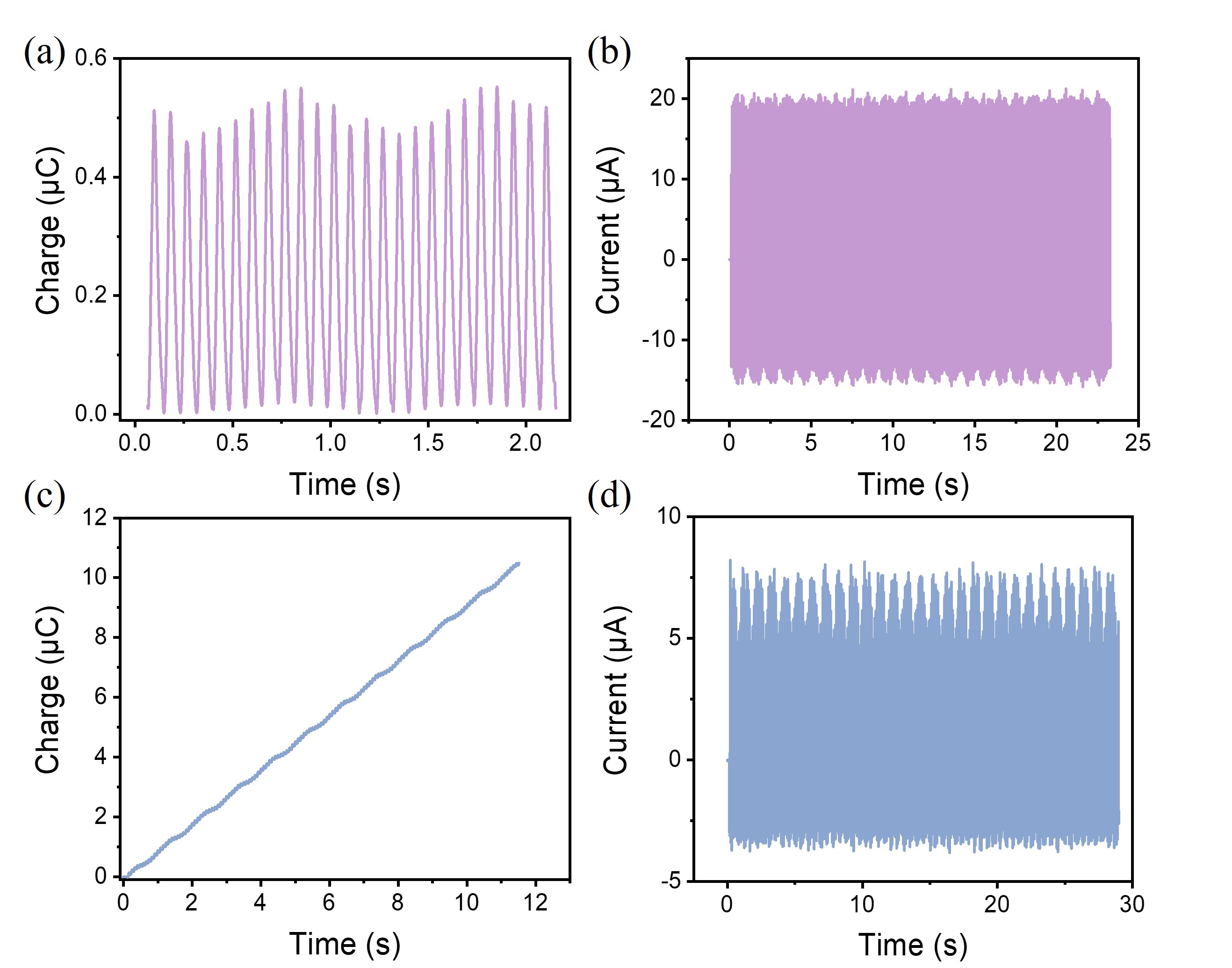


# **Fig. S13** Output performance of the rotary DDO-TENG with 12 pairs of electrodes. **a** Charge transfer of the bottom electrode. **b** Current output of the bottom electrode. **c** Charge transfer of the rotor electrode. **d** Current output of the rotor electrode


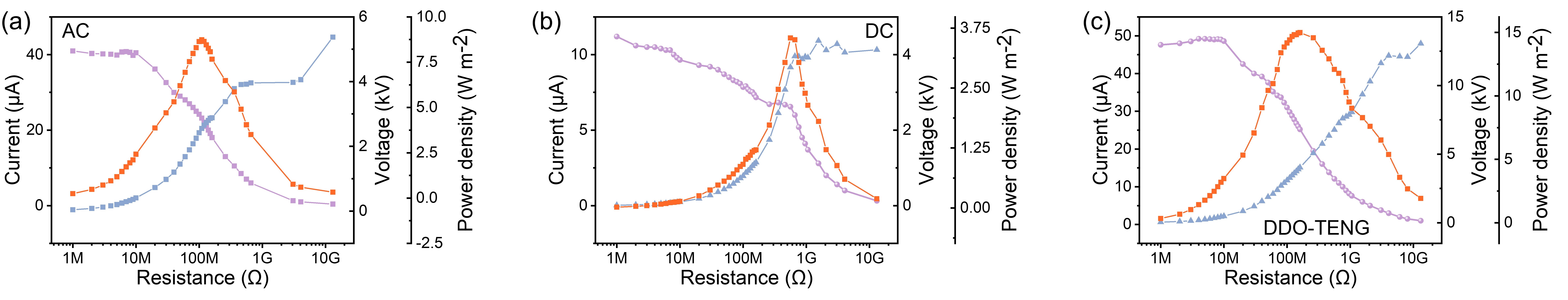


# **Fig.** **S14** The short-circuit current and voltage of the rotary DDO-TENG under different resistances


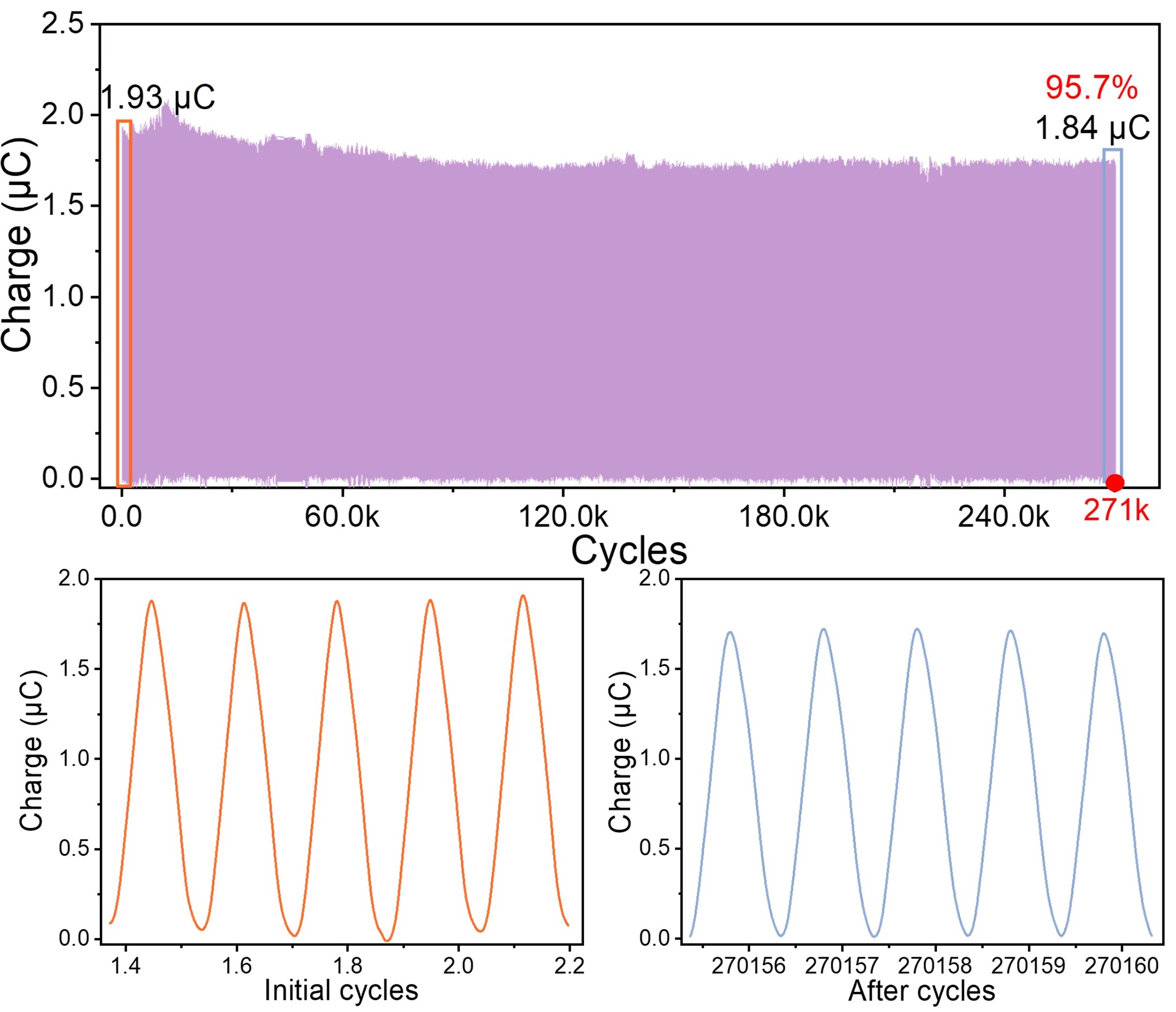


# **Fig.** **S15** The specific transferred charges at the initial stage and after cycling


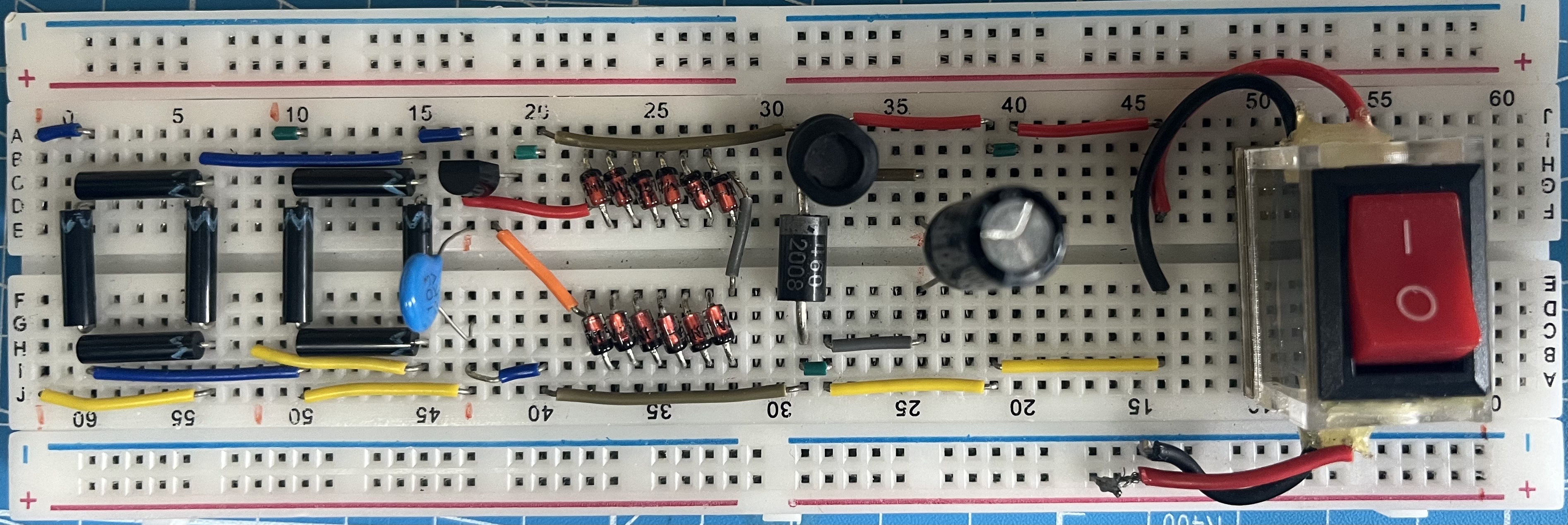


# **Fig. S16** Optical picture of the power management circuit
